# Efficacy of induction regimens for cryptococcal meningitis in HIV-infected adults: a systematic review and network meta-analysis

**DOI:** 10.1038/s41598-021-87726-6

**Published:** 2021-04-21

**Authors:** Chang-Hua Chen, Hua Li, Hsien-Meng Chen, Yu-Min Chen, Yu-Jun Chang, Pao-Yen Lin, Chih-Wei Hsu, Ping-Tao Tseng, Kai-Huang Lin, Yu-Kang Tu

**Affiliations:** 1 Division of Infectious Diseases, Department of Internal Medicine, Changhua Christian Hospital, Changhua, Taiwan; 2 Center for Infection Prevention and Control, Changhua Christian Hospital, Changhua, 500 Taiwan; 3 National Chung Hsing University, Taichung, Taiwan; 4 Rong Hsing Research Center for Translational Medicine, National Chung Hsing University, Taichung, Taiwan; 5 Institute of Epidemiology and Preventive Medicine, College of Public Health, National Taiwan University, Taipei, Taiwan; 6 Department of Pharmacy, Changhua Christian Hospital, Changhua, Taiwan; 7 Epidemiology and Biostatistics Center, Changhua Christian Hospital, Changhua, Taiwan; 8 Department of Psychiatry, Kaohsiung Chang Gung Memorial Hospital, Kaohsiung, Taiwan; 9 Chang Gung University College of Medicine, Kaohsiung, Taiwan; 10 Department of Computer Science and Information Engineering, National Cheng Kung University, Tainan, Taiwan; 11 WinShine Clinics in Specialty of Psychiatry, Kaohsiung City, Taiwan; 12 Division of Critical Care Medicine, Department of Internal Medicine, Changhua Christian Hospital, Changhua, Taiwan; 13 Department of Dentistry, National Taiwan University Hospital, Taipei, Taiwan

**Keywords:** Fungal infection, HIV infections, Meningitis

## Abstract

Cryptococcal meningitis (CM) is the most fatal adult meningitis in patients with human immunodeficiency virus (HIV). There is no conclusive evidence for the superiority of 1-week amphotericin B deoxycholate (AmphB) + flucytosine (5-FC) regimen over other antifungals in the management of HIV patients with CM (HIV–CM patients). We aimed to evaluate the differences in efficacy and tolerability of different antifungal agents in HIV–CM patients by conducting a current network meta-analysis NMA. Overall, 19 randomized controlled trials were included with 2642 participants. A regimen indicated a possibly lower early mortality rate, namely, AmphB + 5-FC + Azole (OR = 1.1E−12, 95% CIs = 1.3E−41 to 0.06) comparing to AmphB + 5-FC. The current NMA provides evidence that AmphB + 5-FC + Azole are superior to all the investigated treatments for induction regimen in HIV–CM patients.

## Introduction

Cryptococcal meningitis (CM) is the most fatal meningitis in adult patients with human immunodeficiency virus (HIV)^1–3^ infection, particularly in those with acquired immunodeficiency syndrome^4^. An effective anti-cryptococcal regimen is needed to treat HIV-associated CM (HIV–CM)^5,6^. HIV–CM treatment can be divided into three phases: induction, consolidation, and maintenance^5,7^. During the induction phase, antifungal treatment needs to decrease cryptococcal burden in the cerebrospinal fluid (CSF) to increase patient survival^5,8^. Amphotericin B deoxycholate (AmphB)-based regimen is widely used during the 2-week induction period for HIV–CM, with fluconazole or flucytosine (5-FC) being used for synergistic effects^5,9^. Research suggests that fluconazole has good tissue penetration^10^; moreover, 5-FC has a fungicidal effect against *Cryptococcus neoformans*^5^. The updated WHO (2018) guidelines for the preferred induction regimens to treat HIV–CM^6^ recommended a change from 2 weeks of AmphB + 5-FC or 2 weeks AmphB + fluconazole into 1 week of AmphB + 5-FC followed by 1 week of fluconazole, and this offers a lower risk of medication toxicity and a reduced risk of nosocomial sepsis^6,11^.

The WHO recommendation was based largely on the findings of meta-analyses reporting that 1 week of AmphB + 5-FC was possibly superior to other regimens^6,11,12^. However, the amount of outcome data used in these analysis was insufficient for direct comparisons between regimens, resulting in an imprecise evaluation of treatment effects^12^. A new study of a novel single-dose 10 mg/kg liposomal AmphB (LipAmphB) showed no statistically significant compared to 14 days of 3 mg/kg/day LipAmphB^13^; furthermore, additional information on this topic has now become available^14^. A network meta-analysis (NMA) showed similar efficacy between the 1-week and 2-week induction regimens of AmphB + 5-FC^12^. In addition, 5-FC is both non-accessible and non-affordable in resource-limited settings that experience heavy cryptococcal burden, such as Africa^11^. Another NMA that compared the efficacy of AmphB + 5-FC and AmphB + fluconazole showed similar outcomes of late mortality rate^15^. These reports demonstrated that the most effective and tolerable induction regimen for HIV–CM has not been completely elucidated as yet. Therefore, we conducted a systematic review and NMA of randomized controlled trials (RCTs) to compare the efficacy and safety of induction regimens of anti-cryptococcal agents in HIV–CM patients.

## Materials and methods

**Figure 1:**
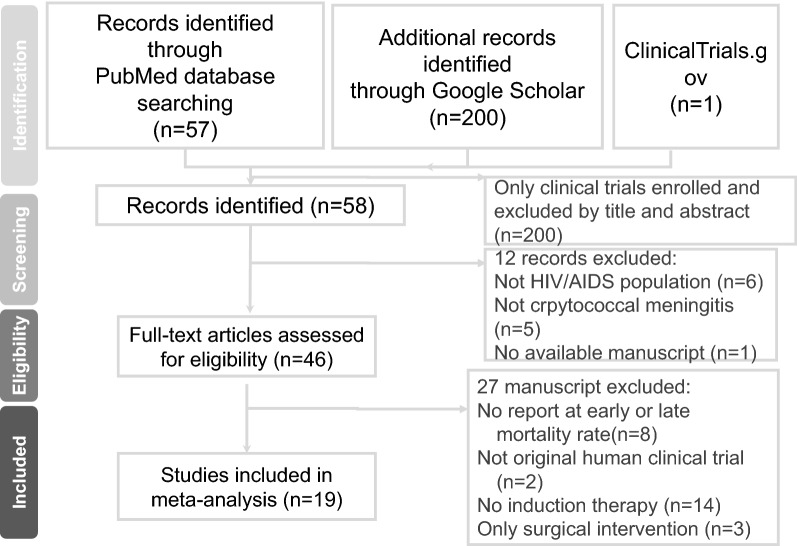
Flowchart of the current network meta-analysis.

A systematic review and NMA were performed to evaluate the effectiveness and safety of different induction regimens in HIV patients with CM (HIV–CM patients). This protocol was approved by the institutional review board of Changhua Christian Hospital (CCH IRB No. 180801). The current study compared the efficacy and safety of induction regimens between AmphB + 5-FC and other evailable regimens in treating HIV–CM patients (Supplementary Table 1). The current NMA was performed according to the preferred reporting items for systematic reviews and meta-analyses (PRISMA) extension guideline for NMAs (Supplementary Table 2, Fig. 1)^16^.

### Search strategy and selection criteria

Several electronic databases were searched from their creation up to Feb 28, 2020: the Cochrane Infectious Diseases Group Trial Register, the Cochrane Central Register of Controlled Trials, MEDLINE (accessed via PubMed). Keywords and search terms used when looking for clinical trials are listed in Supplementary Table 3; the search was not limited to publications in English. However, reviews, correspondence, case reports, expert opinions, and editorials were excluded (Supplementary Table 4).

### Data collection and inclusion criteria

Following the PRISMA guideline^16^, two reviewers (CHC, CYM) screened separately the titles and abstracts of identified articles. Discrepancies or issues of study methodology and quality were resolved by consulting with the third reviewer (CYJ). Studies were included if (1) patients were randomly assigned to different treatments; (2) they were published as full-length articles in peer-reviewed journals, and (3) the efficacy or adverse events of antifungals in treating CM were reported. Excluded studies are listed in the Supplementary Table 4.

### Data extraction

An information-extraction form was created and the following data were collected: (1) details of study design and publication; (2) baseline patient information; (3) the total number of recruited patients; (4) all-cause mortality rates by weeks 2 and 10; (5) mycological suppression; (6) hepatic adverse reaction (Supplementary Tables 5 and 6).

### Outcome definitions

Two primary outcomes and two secondary outcomes were identified. Two primary outcomes were early mortality rate and late mortality rate. The definition of early mortality rate was all-cause mortality rate within 2 weeks of diagnosing CM. The definition of late mortality rate was all-cause mortality rate occurring more than 6 weeks after diagnosing CM. Two secondary outcomes were microbiological eradication and hepatic adverse reaction. The microbiological-eradication outcome was derived from measuring mycological suppression based on quantitative CSF cultures or the rate of change in colony-forming-units (CFU) of fungal cultures during the initial 2 weeks of induction. The mycological suppression improvement was defined as the mycological suppression decline at a rate of ≤ 0.33 log CFU/day or the equal effect during the first 14 days of treatment^17^. The adverse reaction data were derived from the reported rate of liver damage events. The short course of AmphB was defined as being of 1-week duration. The azole regimen defined as fluconazole and voriconazole in current study. The high dose of azole (azole_H) was defined as fluconazole being larger than 800 mg/day^5^. Characteristics of the early- and late-mortality rates of the included studies are listed in the Supplementary Table 5. Characteristics of the microbiological eradication and hepatic adverse reactions in the included studies are shown in the Supplementary Table 6. Supplementary Table 7 details the key findings of the included studies.

### Cochrane risk-of-bias tool and GRADE ratings

Two independent reviewers (CHC, CYM) evaluated the risk of bias for each domain described in the Cochrane risk-of-bias tool^18^. The study evaluated the certainty of the evidence according to the GRADE framework^19^.

### Network meta-analysis

The odds ratio (OR) with 95% confidence interval (CI) was summarized as the effect size for measuring all outcomes. We undertook the frequentist approach to NMA by using the *mvmeta* command^20^ written for the statistical software package Stata (version 16.0, StataCorp LLC, Texas 77845 USA). When the numbers of event were small, we adopted the Bayesian approach by using the software package WinBUGS (version 1.4.3, Medical Research Council Biostatistics Unit, Cambridge, Massachusetts) and R version 3.6.1 (http://www.r-project.org/). Because the pooled estimates of these pairs are different, funnel plots for NMA center the effect size of each pair of treatments. And, we centered the effect sized values according to previous methodology^21^ We evaluated the potential inconsistency between direct and indirect evidence by using the deign-by-treatment interaction model, loop inconsistency model and node-splitting model^22,23^. We also computed the ranking probabilities of treatments which were then summarized by the surface under the cumulative ranking area (SUCRA) ranging from 0 to 1. A treatment with a greater SUCRA value indicates that its efficacy is closer to that of a perfect treatment which is always the best and has a SUCRA value of 1.

### Ethical approval

The study was approved by the institutional review board of Changhua Christian Hospital (CCH IRB No. 180801).

## Results

### Characteristics and description of the included studies

**Figure 2:**
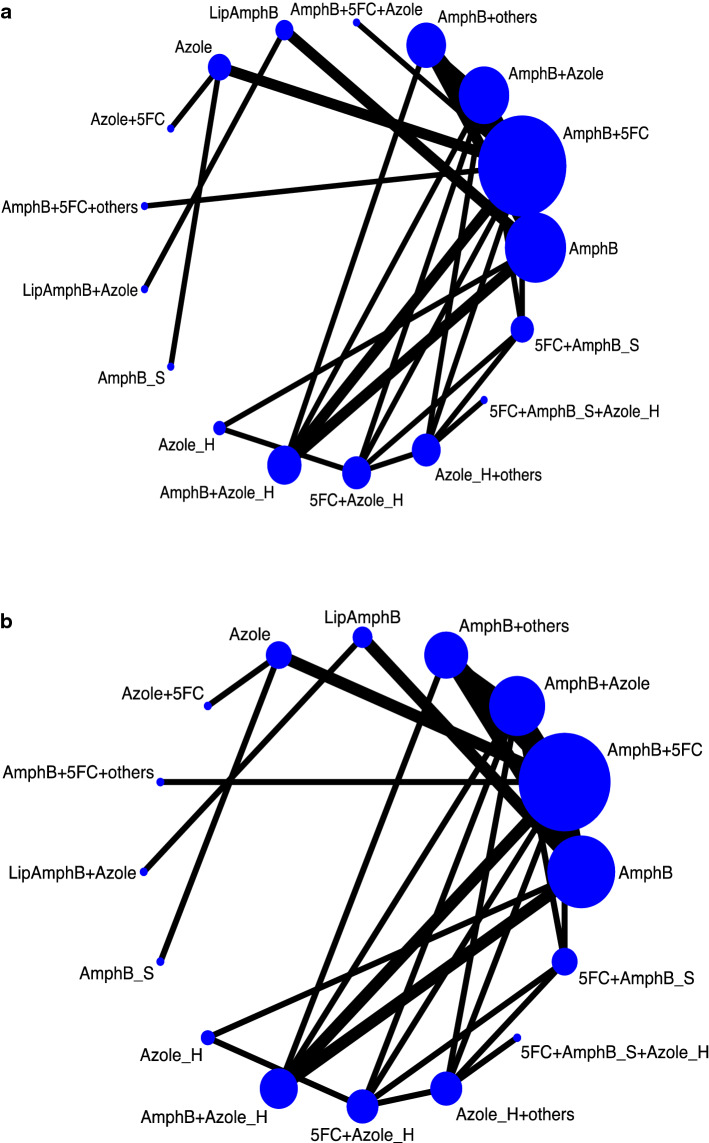
Network structure of network meta-analysis of different outcomes for cryptococcal meningitis in HIV patients. (**a**) Network structure of network meta-analysis of early mortality rate for cryptococcal meningitis in HIV patients. (**b**) Network structure of network meta-analysis of late mortality rate for cryptococcal meningitis in HIV patients.

In total, 46 publications were considered for full-text review, and 27 were excluded (Supplementary Table 3). Finally, 19 articles were included in our NMA (Supplementary Table 5). The quality of the included studies and their risk of bias were rated. Figure 1 depicts the entire geometric distribution of the treatment arms. A total of 2642 participants were included; the baseline characteristics of the included participants are summarized in Supplementary Tables 4 and 5. In brief, 19 studies reported the early-mortality rate and 5 were multi-arm trials; 18 reported the late-mortality rate and 5 were multi-arm trials; 10 reported mycological suppression and 4 were multi-arm trials; and 11 reported hepatic adverse reaction involving 3 multi-arm trials. Figure 2 and Supplementary Figure 1 depict the entire geometric distribution of the treatment arms for four different outcomes.

### Primary outcomes

**Figure 3:**
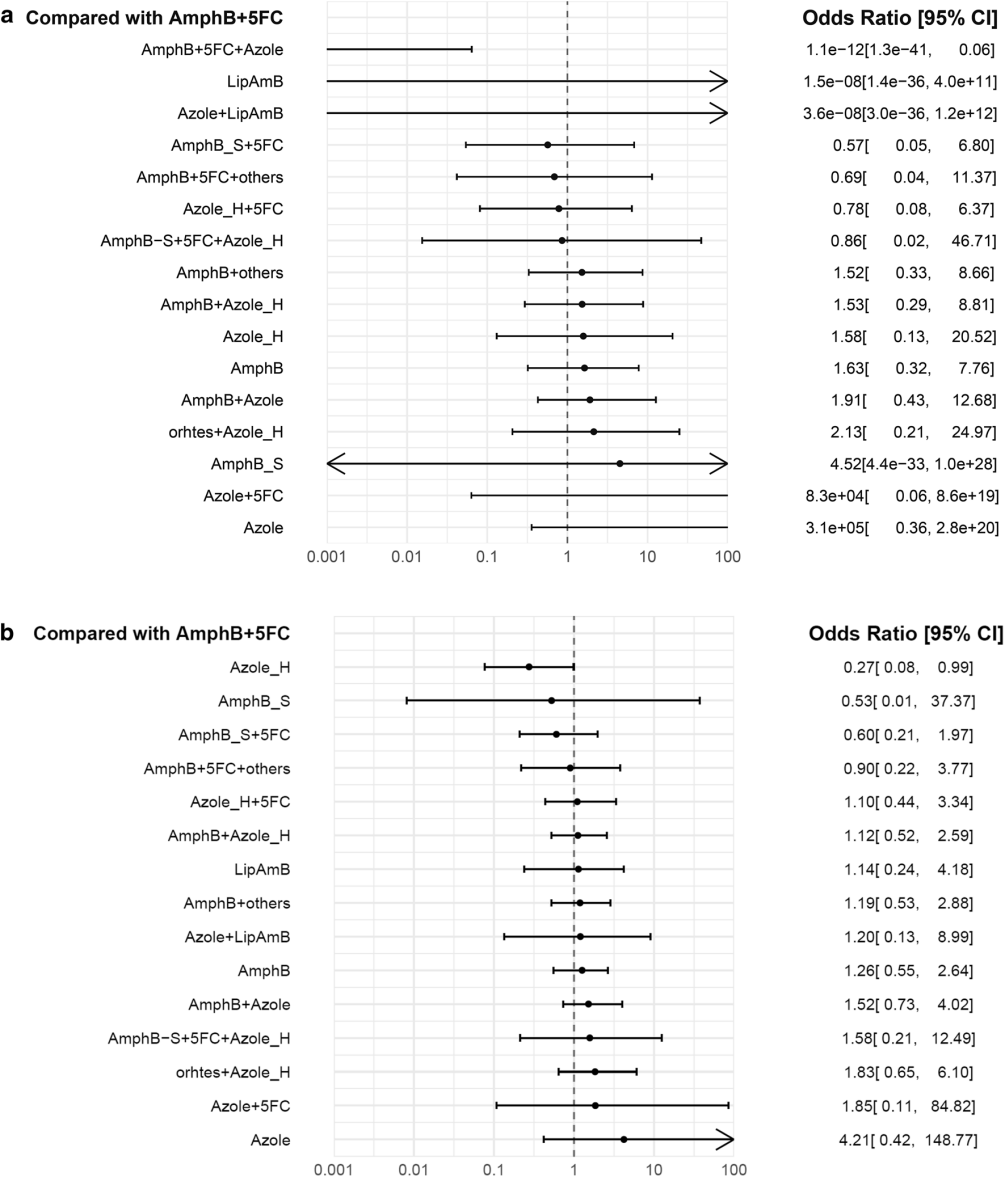
Forest plot of network meta-analysis of two major outcomes for cryptococcal meningitis in HIV patients. (**a**) Forest plot of network meta-analysis of early mortality rate for cryptococcal meningitis in HIV patients. (**b**) Forest plot of network meta-analysis of late mortality rate for cryptococcal meningitis in HIV patients. AmphB, amphotericin B; AmphB_S, short course (only 1 week) amphotericin B; Azole_H, high dose (> 800 mg day); cryptococcal meningitis, CM; fluconazole; 5-FC, flucytosine; human immunodeficiency virus, HIV; LipAmB, liposomal amphotericin B; NMA, network meta-analysis. *Notes*: the left of the null axis at 1 favor the lower mortality rate and those to the right favor the higher mortality rate.

The NMA showed that all the investigated antifungals associated with early mortality rate were similar to those seen in the AmphB + 5-FC-treated participants with CM (Fig. 3a). According to the forest plot, three regimens were possibly related to lower early mortality rate, namely AmphB + 5-FC + Azole (OR = 1.1E−12, 95% CIs = 1.3E−41 to 0.06), liposomal amphotericin B (LipAmphB) (OR = 1.1E−8, 95% CIs = 1.4E−36 to 4.0E−11), and LipAmphB + Azole (OR = 3.6E−8, 95% CIs = 3.0E−36 to 1.12E+12) comparing to AmphB + 5-FC. According to the League table (Supplementary Table 8A), and the SUCRA evaluation (Supplementary Table 9), AmphB + 5-FC + azole was associated with the lowest risk of early mortality rate, followed by short-course AmphB (AmphB_S) and AmphB_S + 5-FC.

### Co-primary outcome: late mortality rate

The NMA revealed that all the investigated antifungals associated with the late mortality rate were similar to the AmphB + 5-FC in participants with CM (Fig. 3b). According to the forest plot, the Azole_H was possibly associated with the lowest late-mortality rate (OR = 0.27, 95% CIs = 0.08–0.99) followed by AmphB_S (OR = 0.53, 95% CIs = 0.01–37.37) and 5-FC + AmphB_S (OR = 0.60, 95% CIs = 0.21–1.97) comparing to AmphB plus 5-FC. According to the league table (Supplementary Table 8B), Azole_H alone presented to higher early mortality rate than most regimens. According to SUCRA (Supplementary Table 9), azole_H was associated with the lowest risk of the late-mortality rate followed by AmphB_S + 5-FC + azole_H.

### Secondary outcomes: mycological suppression and hepatic adverse event rate

The NMA revealed that all the investigated antifungals were associated with mycological suppression similar to the AmphB + 5-FC in participants with CM (Supplementary Figure 2A, Supplementary Table 8C). According to the forest plot, three regimens were possibly related to a higher fungicidal activity, namely AmphB_S + 5-FC (OR = 0.71, 95% CIs = 0.15–4.4), AmphB_S + 5-FC + other (OR = 0.89, 95% CIs = 0.13–6.3), and LipAmphB (OR = 0.96, 95% CIs = 0.11–6.1) compared to AmphB + 5-FC. In contrast, two regimens were possibly related to a poorly fungicidal activity, namely azole_H (OR = 3.8, 95% CIs = 0.62–23) and azole_H + others (OR = 2.2, 95% CIs = 0.46–14), According to SUCRA (Supplementary Table 9), others + azole_H was associated with the lowest microbiological activity followed by AmphB + 5-FC + others and an AmphB + 5-FC.

The NMA revealed that all the investigated antifungals were associated with a hepatic-adverse-event rate similar to that of AmphB and 5-FC in participants with CM (Supplementary Figure 2B, Supplementary Table 8D). According to the forest plot, the azole regimen was probably associated with the lowest adverse event (OR = 0.075, 95% CIs =  < 0.001–1.6) followed by LipAmphB (OR = 0.50, 95% CIs = 0.018–11) and azole_H + 5-FC (OR = 0.55, 95% CIs = 0.059–3.9) comparing to AmphB with 5-FC.

### Risk of bias, publication bias, inconsistency assessment, and GRADE ratings

We found that 61.7%, 14.3%, and 24.0% of the enrolled 19 studies showed a low, unclear, and high risk of bias, respectively. Unclear reporting of the allocation procedures and blinding of the participants or research personnel was the most often encountered reason for the high risk of bias (Supplement Figure 3). The overall quality of direct and indirect evidence in the overall NMA was low to medium based on GRADE evaluation (Supplementary Figure 3).

We found no evidence of inconsistencies by using either loop-specific approach, node-splitting approach, or design-by-treatment approach (Supplementary Table 10).

Funnel plots of the publication bias (Supplement Figure 4) showed general symmetry. No significant publication bias among the included studies was evaluated by Egger’s test. Because intercept significantly is close zero, small study bias is not significant.

## Discussion

Our NMA summarizes the current evidence on the efficacy and tolerability of the induction therapy for individual antifungals in HIV–CM patients. And our results showed that AmphB + 5-FC + Azole as induction regimen yielded a lower mortality rate than other antifungal regimens. All the investigated antifungals were associated with a similar early mortality rate and also with a similar late mortality rate in HIV–CM patients. Based on mycological suppression, the azole_H alone was not recommended to be induction regimen in HIV–CM patients due to poor fungicidal activity. In summary, AmphB + 5-FC + Azole are superior to all investigated regimens for induction and the azole_H alone was not recommended to be induction regimen in HIV–CM patients due to poor fungicidal activity in HIV–CM patients.

Our NMA also found that the AmphB + 5-FC + Azole are superior to all the investigated treatments for induction regimens in HIV–CM patients. In contrast to Tenforde et al., our study did not found that 1-week AmphB + 5-FC-based therapy was superior to other regimens used to treat HIV–CM^11^. As shown in a previous meta-analysis^12^, the combination of AmphB + 5-FC + azole showed mostly significantly lower early mortality rate (Fig. 1), but, the result came from only a single study^24^. The evidence is not conclusive due to the small sample bias. In addition, there was no statistically significant difference in mortality rates and hepatic adverse events between AmphB + azole_H and AmphB + 5-FC for HIV–CM patients. Briefly, our study shows 1-week AmphB + 5-FC remains the best preferred regimen than others in HIV–CM patients.

The goal of treating CM is to attain cryptococcal eradication and to protect from neurological damage^25,26^. Mycological suppression could achieve fungal eradication in CSF as a prognostic factor. In previous studies, mycological suppression was independently associated with mortality^17,27,28^. Our result is similar to a previous report^14^ showing no statistically significant difference in MS between the investigated antifungals. Our current NMA revealed that all the investigated antifungals were associated with a similar mycological suppression compared to AmphB + 5-FC in participants with mycological suppression, but the azole_H alone presented with poorly fungicidal activity (OR = 3.8, 95% CIs = 0.62–23). The results of our analysis is in line with those of a previous report^29^ that the azole_H alone was not recommended to be an induction regimen in HIV–CM patients due to poor fungicidal activity. Due to disconnection of network from the reporting of mycological suppression events, the secondary outcome needs further evaluation to elucidate this viewpoint.

Anti-fungal agents have high toxicity and cause hepatotoxicity, although the effectiveness of AmphB for HIV–CM patients is outstanding. The administration of azole with AmphB is recommended for the induction treatment of CM^30–32^. The most frequent hepatic adverse events were due to an increased hepatic injury related to azole. Guidelines issued in 2010 recommended AmphB plus fluconazole (800 mg/day) as the induction therapy^5^. According to the current NMA, azole_H did not achieve a significantly cumulative hepatic toxicity effect. Moreover, AmphB plus fluconazole (800 mg/day) could be adopted as the standard induction antifungal regimen after Cryptococcal Optimal ART Timing Trial for HIV–CM patients^14^.

## Limitations

There are several limitations to be acknowledged in the current NMA. First, some data analyzed in this study were limited by under-powered statistics, namely in heterogeneity between and within studies and a small number of trials for some treatment arms. Second, in the current NMA, we did not exclude trials with small case numbers in both the intervention and control arms because most RCTs had zero of relapse cases; this yielded a relatively large confidence (or credible) intervals for some treatment comparisons, although we also adopted the Bayesian model to obtain more robust estimates^33,34^. Lastly, in spite of comparing different antifungals in our NMA, future large RCTs are required to evaluate the effectiveness and safety of different induction regimens to determine the best regimen for the management of HIV–CM patients.

## Conclusions

Our NMA provides synthesized current evidence on the efficacy and safety of individual antifungals in patients with HIV–CM for early mortality. We found that AmphB + 5-FC + Azole are superior to all the investigated treatments as induction regimens for HIV–CM. Our NMA contributes compelling evidence the impactful evidence with AmphB + 5-FC + Azole for treating HIV–CM to alleviate the burden of CM. Furthermore, azole_H alone is not recommended as an induction regimen for HIV–CM owing to its poor fungicidal activity. However, given the small number of studies included in the current analysis, future large-scale RCTs focusing on the efficacy of different dosages and treatment durations of antifungals in patients with HIV–CM should be conducted to support or refute the results of the current NMA.

## Supplementary Information

Supplementary Tables.

Supplementary Figures.
