# Supplementary material for: Efficacy of induction regimens for cryptococcal meningitis in HIV-infected adults: a systematic review and network meta-analysis
Source: Sci Rep. 2021 Apr 21;11:8565. doi: 10.1038/s41598-021-87726-6 (PMC8060388; doi:10.1038/s41598-021-87726-6)
Supplement: Supplementary file 1 — Supplementary Tables. [file 41598_2021_87726_MOESM1_ESM.docx]

Category of paper: Review

Article title: Re-appraisal of effective, and tolerable induction regimens for managing cryptococcal meningitis in HIV-infected adults: a systematic review and network meta-analysis

Authors details

Names of authors:

Chang-Hua Chen ^a,b,c,d^, Hua Li^e, @^, Hsien-Meng Chen^a^, Yu-Min Chen ^f^, Yu-Jun Chang ^g^, Pao-Yen Lin ^h, i,^ Chih-Wei Hsu ^h, j^, Ping-Tao Tseng ^k^, Kai-Huang Lin^l,^ Yu-Kang Tu ^m,n,*^

Affiliation:

^a^ Division of Infectious Disease, Department of Internal Medicine, Changhua Christian Hospital, Changhua, 500, Taiwan;

^b^ Center for Infection Prevention and Control, Changhua Christian Hospital, Changhua, 500, Taiwan ;

^c^ Ph.D. Program in Translational Medicine, National Chung Hsing University, Taichung County, 402, Taiwan ;

^d^ Rong Hsing Research Center For Translational Medicine, National Chung Hsing University, Taichung County, 402, Taiwan ;

^e^ Institute of Epidemiology & Preventive Medicine, College of Public Health, National Taiwan University, Taipei, Taiwan

^f^ Department of Pharmacy, Changhua Christian Hospital, Changhua, Taiwan

^g^ Epidemiology and Biostatistics Center, Changhua Christian Hospital, Changhua, Taiwan

^h^ Department of Psychiatry, Kaohsiung Chang Gung Memorial Hospital, Kaohsiung, Taiwan

^i^ Chang Gung University College of Medicine, Kaohsiung, Taiwan

^j^ Department of Computer Science and Information Engineering, National Cheng Kung University, Tainan, Taiwan

^k^ WinShine Clinics in Specialty of Psychiatry, Kaohsiung City, Taiwan

^l^ Division of Critical Care Medicine, Department of Internal Medicine, Changhua Christian Hospital, Changhua, 500, Taiwan;

^m^Institute of Epidemiology & Preventive Medicine, College of Public Health, National Taiwan University, Taipei, Taiwan

^n^ Department of Dentistry, National Taiwan University Hospital, Taipei, Taiwan

^@^: equal contribution

Corresponding author: Yu-Kang Tu, Institute of Epidemiology & Preventive Medicine, College of Public Health, National Taiwan University, Taipei, Taiwan; e-mail: yukangtu@ntu.edu.tw

Supplementary Tables

Supplementary Table 1 Definition of the PICO in current study

Supplementary Table 2 systematic reviews and meta-analyses (PRISMA) extension guideline

Supplementary Table 3 Complete search strategies through PubMed database searching

Supplementary Table 4 Excluded studies and reasons

Supplementary Table 5 Characteristics of the early and late mortality features among included studies

Supplementary Table 6 Characteristics of the microbiological eradication and hepatic adverse reaction among included studies

Supplementary Table 7 Characteristics of key findings in the included studies

Supplementary Table 8A League table of the early mortality

Supplementary Table 8B League table of the late mortality

Supplementary Table 8C League table of mycological suppression

Supplementary Table 8D League table of the hepatic adverse reaction

Supplementary Table 9 SUCRA of the outcomes of individual interventions

Supplementary Table 10 Inconsistency of different intervention

Supplementary Table 1 Definition of the PICO in current study

| Patient | Human immunodeficiency virus infected patients associated with Cryptococcal meningitis(HIV-CM) |
| --- | --- |
| Intervention | Amphotericin B deoxycholate with flucytosine |
| Comparator | Other available induction regimen for treating HIV-CM, including:  AmphB 0.3m/k/d + 5FC 150m/k/d (6 wk)  AmphB 0.3m/k/d + 5FC 150m/k/d + Itra 400m/d (6 wk)  AmphB 0.3m/k/d +5FC 150m/k/d (6 wk)  AmphB 0.7-1m/k/d + 5FC 100m/k/d (2 wk)  AmphB 0.7-1m/k/d + Fluc 800m/d (2 wk)  AmphB 0.7-1m/k/d + Voriconazole 600m/d (2 wk)  AmphB 0.7m/k/d (1 week), then 0.7mg/kd/3d (9 wk) + 5FC 150m/k/d (10 wk)  AmphB 0.7m/k/d (2 wk)  AmphB 0.7m/k/d (3 wk)  AmphB 0.7m/k/d + Rifampin 600m/d (2 wk)  AmphB 0.7m/k/d +5FC 100m/k/d (2 wk)  AmphB 0.7m/k/d +5FC 100m/k/d +Fluc 400m/d (2 wk)  AmphB 0.7m/k/d +Fluc 400m/d (2 wk)  AmphB 0.7mg/kg (2 wk)  AmphB 0.7mg/kg + Fluc 400 (2 wk)  AmphB 0.7mg/kg + Fluc 800 (2 wk)  AmphB 1m/k/d (1 week)  AmphB 1m/k/d (1 week) + Fluc 1200m/d (2 wk)  AmphB 1m/k/d (1 week) + Fluc 1200m/d (2 wk) + 5FC 100m/k/d (2 wk)  AmphB 1m/k/d (4 wk)  AmphB 1m/k/d + 5FC 100m/k/d (1 wk), the Fluc 1200 m/d (1wk)  AmphB 1m/k/d + 5FC 100m/k/d (2 wk)  AmphB 1m/k/d + Fluc 1200m/d (1 wk), then Fluc 1200m/d (1 wk)  AmphB 1m/k/d + Fluc 1200m/d (2 wk)  AmphB 1m/k/d + Fluc 800m/d (2 wk)  Fluc 1200m/d (2 wk)  Fluc 1200m/d + 5FC 100m/k/d (2 wk)  Fluc 200m/d (2 wk)  Fluc 200m/d + 5FC 150m/k/d (2 wk)  Fluc 400m/d (10 wk)  IFNg 100ug 2x + AmphB 1m/k/d + 5FC 100m/k/d (2 wk)  Itra 400m/d (6 wk)  L-AmphB 10 mg/kg on D 1 & 5 mg/kg on day 3 (2 doses)+ Fluc 1200 m/d (2 wk)  L-AmphB 10 mg/kg on D 1 and 5 mg/kg on D 3 and 7 (3 doses)+ Fluc 1200 m/d (2 wk)  L-AmphB 10 mg/kg on D1 (single dose) + Fluc 1200 m/d (2 wk)  L-AmphB 3 m/k/d (2 wk)  Lip AmphB 4m/k/d (3 wk) |
| Outcome | Outcomes measures include as follows:  Early-Mortality Rates  Late-Mortality Rates  Microbiological Eradication Events  Hepatic Adverse Events |

Abbreviation: 5FC, flucytosine; AmphB, amphotericin B; AmphB_S, short course (only 1week) amphotericin B; Fluc, fluconazole; Fluc_H, high dose (fluconazole>800 mg/day); IFNg, Interferon gamma; Itra, itraconazole; Lip AmphB, liposomal amphotericin B; m/g, mg/day; m/g/k, mg/kg/day;wk, week.

Supplementary Table 2 systematic reviews and meta-analyses (PRISMA) extension guideline

| **Section/Topic** | **#** | **Checklist Item** | **Reported on Page #** |
| --- | --- | --- | --- |
| **TITLE** | | | |
| Title | 1 | Identify the report as a systematic review, meta-analysis, or both. | 1 |
| **ABSTRACT** | | | |
| Structured summary | 2 | Provide a structured summary including, as applicable: background; objectives; data sources; study eligibility criteria, participants, and interventions; study appraisal and synthesis methods; results; limitations; conclusions and implications of key findings; systematic review registration number. | 2-3 |
| **INTRODUCTION** | | | |
| Rationale | 3 | Describe the rationale for the review in the context of what is already known. | 4-5 |
| Objectives | 4 | Provide an explicit statement of questions being addressed with reference to participants, interventions, comparisons, outcomes, and study design (PICOS). | 5 |
| **METHODS** | | | |
| Protocol and registration | 5 | Indicate if a review protocol exists, if and where it can be accessed (e.g., Web address), and, if available, provide registration information including registration number. | - |
| Eligibility criteria | 6 | Specify study characteristics (e.g., PICOS, length of follow-up) and report characteristics (e.g., years considered, language, publication status) used as criteria for eligibility, giving rationale. | 5 |
| Information sources | 7 | Describe all information sources (e.g., databases with dates of coverage, contact with study authors to identify additional studies) in the search and date last searched. | 5-6 |
| Search | 8 | Present full electronic search strategy for at least one database, including any limits used, such that it could be repeated. | 6 |
| Study selection | 9 | State the process for selecting studies (i.e., screening, eligibility, included in systematic review, and, if applicable, included in the meta-analysis). | 5-6 |
| Data collection process | 10 | Describe method of data extraction from reports (e.g., piloted forms, independently, in duplicate) and any processes for obtaining and confirming data from investigators. | 6 |
| Data items | 11 | List and define all variables for which data were sought (e.g., PICOS, funding sources) and any assumptions and simplifications made. | 6-7 |
| Risk of bias in individual studies | 12 | Describe methods used for assessing risk of bias of individual studies (including specification of whether this was done at the study or outcome level), and how this information is to be used in any data synthesis. | 8 |
| Summary measures | 13 | State the principal summary measures (e.g., risk ratio, difference in means). | 8-9 |
| Synthesis of results | 14 | Describe the methods of handling data and combining results of studies, if done, including measures of consistency (e.g., I^2^) for each meta-analysis. | 8-9 |

Supplementary Table 3 Complete search strategies through PubMed database searching

|  | Search term | Paper number |
| --- | --- | --- |
| #1 | meningitis or meningitis or meningitides or central nervous system infections or central nervous system fungal infections, fungal meningitis | 173210 |
| #2 | cryptococc* | 17756 |
| #3 | #1 and #2 | 4574 |
| #4 | # 3 and (therapy or treatment) | 2900 |
| #5 | azole* or fluconazole or amphotericin* or flucytosine or itraconazole or *conazole or 5-FC | 994 |
| #6 | #4 and #5 | 1402 |
| #7 | hiv or hiv-1* or hiv-2* or hiv1 or hiv2 or (hiv near infect*) or (human immunodeficiency virus) or (human immunedeficiency virus) or (human immune-deficiency virus) or (human immuno-deficiency virus) or (human immune deficiency virus) or (human immuno deficiency virus) or (acquired immunodeficiency syndrome) or (acquired immunedeficiency syndrome) or (acquired immuno-deficiency syndrome) or (acquired immune-deficiency syndrome) or (acquired immun* deficiency syndrome) | 810 |
| #8 | #6 and #7 and ( *trial) | 57 |

Supplementary Table 4: Excluded studies and reasons

| Study | Reason for exclusion |
| --- | --- |
| Larsen et al. 1990 | Small study of 20 participants comparing fluc to AmphB and 5FC, which was excluded for several reasons: did not do intention-to-treat analysis; excluded significant number of participants (6/26) from analysis after enrolment; enrolled non-HIV-positive patient; final comparison groups poorly matched on CD4 count |
| Bozzette et al. 1991 | RCT with participants randomized to maintenance therapy |
| Saag et al. 1991 | RCT that included patients with relapsed cryptococcal meningitis |
| de Gans et al. 1992 | Small study of 25 participants randomized to itrac versus AmphB and 5FC for induction therapy where 2/14 participants in itra group crossed over within 4 hours and an additional 2 participants were lost to follow-up |
| Powderly et al. 1992 | RCT of secondary prophylaxis for prevention of recurrent cryptococcal meningitis |
| Powderly et al. 1995 | RCT of primary prophylaxis for fungal infections |
| Joly_a et al. 1996 | Included patients with relapsed cryptococcal meningitis |
| Joly_b et al. 1996 | Included patients with relapsed cryptococcal meningitis |
| Sharkey et al. 1996 | Small RCT comparing AmphB with different doses of liposomal AmphB in which CD4 count and other baseline characteristics were not well matched between groups |
| Chotmongkol et al. 1997 | RCT of consolidation therapy with itra versus no therapy after CSF culture clearance |
| Saag et al. 1999 | RCT of consolidation therapy for cryptococcal meningitis |
| Chariyalertsak et al. 2002 | RCT with participants randomized to consolidation therapy, not induction therapy |
| Newton et al. 2002 | Small RCT of adjunctive acetazolamide that did not limit inclusion to patients with HIV-associated cryptococcal meningitis |
| Mootsikapun et al. 2003 | RCT with participants randomized to consolidation therapy, not induction therapy |

Abbreviation: 5FC, flucytosine; AmphB, amphotericin B; RCT, randomized clinical trial, Itra, itraconazole; Fluc, fluconazole

Supplementary Table 5: Characteristics of the early and late mortality features among included studies

| **Author , Year, Location** | **Study Design/Number Rand** | **Age(years-old) /Male(%)/CD_4_T-cell count(/μL)** | **Therapeutic Arms** | **Total Recruited** | **Early mortality (2-wk)** | **Late mortality (6-12 wk)** |
| --- | --- | --- | --- | --- | --- | --- |
| Larsen et al. 1990, USA | Prosp, OL, Rand | NM/NM/NM | Fluc 400m/d (10 wk) | 14 | NR | 10wk(4/14) |
|  |  |  | AmphB 0.7m/k/d (1 week), then 0.7mg/kd/3d (9 wk) + 5FC 150m/k/d (10 wk) | 6 | NR | 10wk(0/6) |
| de Gans et al. 1992,Netherlands | Prosp, OL, Rand | meD 34/NM/< 200 | Itra 400m/d (6 wk) | 14 | 43844 | 6wk(1/14) |
|  |  |  | AmphB 0.3m/k/d +5FC 150m/k/d (6 wk) | 11 | 0/11 | 6wk(1/11) |
| van der Horst et al. 1997,USA | Prosp, DB, Rand | meD 37/43.6/NM | AmphB 0.7m/k/d +5FC 150m/k/d (2 wk) | 202 | 11/202 | 10wk(14/202) |
|  |  |  | AmphB 0.7m/k/d +placebo (2 wk) | 179 | 10/179 | 10wk(12/179) |
| Leenders et al. 1997,Netherlands, Australia | Prosp, OL,Rand | meD 41 | AmphB 0.7m/k/d (3 wk) | 13 | 0/13 | 10wk (1/13) |
|  |  | /NM/NM | Lip AmphB 4m/k/d (3 wk) | 15 | 0/15 | 10wk (2/15) |
| Chotmongkol et al. 1997,Thailand | Prosp, OL,Rand | meD, 29/ 92 /meA, 22.3 | AmphB 0.3m/k/d + 5FC 150m/k/d + Itra 400m/d (6 wk) | 50 | 0/50 | NR |
|  |  |  | AmphB 0.3m/k/d + 5FC 150m/k/d (6 wk) | 50 | 4/50 | NR |
| Mayanja-Kizza et al. 1998,Uganda | Prosp, OL, Rand | meD 34 /43 /meA 77 | Fluc 200m/d + 5FC 150m/k/d (2 wk) | 30 | 43946 | 8 wk(11/25) |
|  |  |  | Fluc 200m/d (2 wk) | 28 | 44129 | 8 wk(16/25) |
| Brouwer et al. 2004,Thailand | Prosp, OL,Rand | meD 33 /60 | AmphB 0.7m/k/d (2 wk) | 16 | 43877 | 10wk (3/16) |
|  |  | /meD 9 | AmphB 0.7m/k/d +5FC 100m/k/d (2 wk) | 15 | 43845 | 10wk (1/15) |
|  |  |  | AmphB 0.7m/k/d +Fluc 400m/d (2 wk) | 16 | 43967 | 10wk (7/16) |
|  |  |  | AmphB 0.7m/k/d +5FC 100m/k/d +Fluc 400m/d (2 wk) | 16 | 43846 | 10wk (3/16) |
| Chotmongkol et al. 2005,Thailan | Prosp, OL,Rand | meA, 30 /, | AmphB 0.7m/k/d (2 wk) | 20 | 0/20 | 10wk (2/20) |
|  |  | 60/NM | AmphB 0.7m/k/d + Rifampin 600m/d (2 wk) | 20 | 43971 | 10wk (6/20) |
| Bicanic et al. 2007,South Africa | Prosp, OL,observational | meA, 34/ 22/NM | AmphB 1m/k/d (1 week) | 49 | NR | 10wk(16/48) |
|  |  |  | Fluc 400m/d (10 wk) | 5 | NR | 10wk (3/4) |
| Pappas et al. 2009,USA, Thailand | Prosp, OL,Rand | meA 37 | AmphB 0.7mg/kg (2 wk) | 47 | 3/47 | 10wk (7/47) |
|  |  | /65/meD 18 | AmphB 0.7mg/kg + Fluc 400 (2 wk) | 48 | 2/48 | 10wk (6/48) |
|  |  |  | AmphB 0.7mg/kg + Fluc 800 (2 wk) | 48 | 1/48 | 10wk (7/48) |
| Hamill et al. 2010,USA, Canada | Prosp,DB,Rand | meA, 38 / 88/NM | AmphB 0.7m/k/d (2 wk) | 87 | NR | 10wk(10/87) |
|  |  |  | Lip AmphB 3m/k/d (2 wk) | 180 | NR | 10wk(21/180) |
| Nussbaum et al. 2010,Malawi | Prosp, OL, Rand | meD 36 /66 | Fluc 1200m/d (2 wk) | 20 | 44031 | 10wk(11/19) |
|  |  | /meD 21 | Fluc 1200m/d + 5FC 100m/k/d (2 wk) | 21 | 43863 | 10wk (9/21) |
| Loyse et al. 2012,South Africa | Prosp, OL, Rand | meD 34 /49 /meD 37 | AmphB 0.7-1m/k/d + 5FC 100m/k/d (2 wk) | 20 | 43850 | 10wk (6/20) |
|  |  |  | AmphB 0.7-1m/k/d + Fluc 800m/d (2 wk) | 45 | 7/45 | 10wk(13/43) |
|  |  |  | AmphB 0.7-1m/k/d + Voriconazole 600m/d (2 wk) | 13 | 43843 | 10wk (3/12) |
| Jarvis et al. 2012,South Africa | Prosp, OL, Rand | meD 32 /44 /meD 27 | AmphB 1m/k/d + 5FC 100m/k/d (2 wk) | 31 | 6/31 | 10wk(10/31) |
|  |  |  | IFNg 100ug 2x + AmphB 1m/k/d + 5FC 100m/k/d (2 wk) | 57 | 8/57 | 10wk(17/57) |
| Jackson et al. 2012,Malawi | Prosp, OL, Rand | meD 35 /65 /meD 41 | AmphB 1m/k/d (1 week) + Fluc 1200m/d (2 wk) | 20 | 43941 | 10wk (7/20) |
|  |  |  | AmphB 1m/k/d (1 week) + Fluc 1200m/d (2 wk) + 5FC 100m/k/d (2 wk) | 20 | 43881 | 10wk (6/19) |
| Day et al. 2013,Vietnam | Prosp, OL ,Rand | meD 28 /82 | AmphB 1m/k/d (4 wk) | 99 | 25/99 | 10wk(44/99) |
|  |  | /meD 18 | AmphB 1m/k/d + 5FC 100m/k/d (2 wk) | 100 | 15/100 | 10wk(30/100) |
|  |  |  | AmphB 1m/k/d + Fluc 800m/d (2 wk) | 99 | 20/99 | 10wk(33/99) |
| Molloy et al. 2018, United Kingdom | Prosp, OL, Rand | meD 36 /53/meD 25 | AmphB 1m/k/d + 5FC 100m/k/d (2 wk) | 115 | 24/115 | 10wk (44/115) |
|  |  |  | AmphB 1m/k/d + Fluc 1200m/d (2 wk) | 114 | 25/114 | 10wk(47/114) |
|  |  |  | AmphB 1m/k/d + 5FC 100m/k/d (1 wk), the Fluc 1200 m/d (1wk) | 113 | 13/113 | 10wk(27/113) |
|  |  |  | AmphB 1m/k/d + Fluc 1200m/d (1 wk), then Fluc 1200m/d (1 wk) | 111 | 36/111 | 10wk(54/111) |
|  |  |  | oral Fluc 1200 m/d+5FC 100 m/k/d (2 wk) | 225 | 41/225 | 10wk(79/225) |
| Jarvis et al. 2018,Botswana and Tanzania | Prosp, OL, Rand | meD 38 /54 /meD 32 | L-AmphB 10 mg/kg on D1 (single dose) + Fluc 1200 m/d (2 wk) | 18 | 43879 | 10wk(4/18) |
|  |  |  | L-AmphB 10 mg/kg on D 1 & 5 mg/kg on day 3 (2 doses)+ Fluc 1200 m/d (2 wk) | 20 | 43910 | 10wk (3/20) |
|  |  |  | L-AmphB 10 mg/kg on D 1 and 5 mg/kg on D 3 and 7 (3 doses)+ Fluc 1200 m/d (2 wk) | 20 | 43971 | 10wk(10/20) |
|  |  |  | L-AmphB 3 m/k/d (2 wk) | 21 | 2/2 | 10wk(6/21) |
| NCT00885703 | Prosp Rand | NM/NM/NM | AmphB | 108 | 17/108 | 10wk(30/108) |
|  |  |  | Fluc_High | 46 | 4/46 | 10wk (5/46) |

Abbreviation: 5FC , flucytosine; AmphB, amphotericin B; AmphB_S, short course (only 1week) amphotericin B; Fluc, fluconazole; Fluc_H, high dose ( fluconazole > 800 mg/day ); IFNg, Interferon gamma; Itra, itraconazole; Lip AmphB, liposomal amphotericin B; m/g, mg/day; m/g/k, mg/kg/day; meA, mean; meD, median; OL, open label; Prosp, Prospective; wk, week.

Supplementary Table 6: Characteristics of the microbiological eradication and hepatic adverse reaction among included studies

| **Author , Year, Location** | **Study Design/Number Rand** | **Age(years-old) /Male(%)/CD_4_T-cell count(/μL)** | **Therapeutic Arms** | **Total Recruited** | **microbiological eradicatiob** | **hepatic adverse reaction** |
| --- | --- | --- | --- | --- | --- | --- |
| Larsen et al. 1990, USA | Prosp, OL, Rand | NM/NM/NM | Fluc 400m/d (10 wk) | 14 | NR | 5/14 |
|  |  |  | AmphB 0.7m/k/d (1 week), then 0.7mg/kd/3d (9 wk) + 5FC 150m/k/d (10 wk) | 6 | NR | 5/6 |
| de Gans et al. 1992,Netherlands | Prosp, OL, Rand | meD 34/NM/< 200 | Itra 400m/d (6 wk) | 14 | NR | NR |
|  |  |  | AmphB 0.3m/k/d +5FC 150m/k/d (6 wk) | 11 | NR | NR |
| van der Horst et al. 1997,USA | Prosp, DB, Rand | meD 37/43.6/NM | AmphB 0.7m/k/d +5FC 150m/k/d (2 wk) | 202 | 4/151 | NR |
|  |  |  | AmphB 0.7m/k/d +placebo (2 wk) | 179 | 8/155 | NR |
| Leenders et al. 1997,Netherlands, Australia | Prosp, OL,Rand | meD 41 | AmphB 0.7m/k/d (3 wk) | 13 | 2/13 | 1/13 |
|  |  | /NM/NM | Lip AmphB 4m/k/d (3 wk) | 15 | 1/15 | 0/15 |
| Chotmongkol et al. 1997,Thailand | Prosp, OL,Rand | meD, 29/ 92 /meA, 22.3 | AmphB 0.3m/k/d + 5FC 150m/k/d + Itra 400m/d (6 wk) | 50 | NR | NR |
|  |  |  | AmphB 0.3m/k/d + 5FC 150m/k/d (6 wk) | 50 | NR | NR |
| Mayanja-Kizza et al. 1998,Uganda | Prosp, OL, Rand | meD 34 /43 /meA 77 | Fluc 200m/d + 5FC 150m/k/d (2 wk) | 30 | 11/25 | NR |
|  |  |  | Fluc 200m/d (2 wk) | 28 | 16/25 | NR |
| Brouwer et al. 2004,Thailand | Prosp, OL,Rand | meD 33 /60 | AmphB 0.7m/k/d (2 wk) | 16 | 3/16 | NR |
|  |  | /meD 9 | AmphB 0.7m/k/d +5FC 100m/k/d (2 wk) | 15 | 1/15 | NR |
|  |  |  | AmphB 0.7m/k/d +Fluc 400m/d (2 wk) | 16 | 7/16 | NR |
|  |  |  | AmphB 0.7m/k/d +5FC 100m/k/d +Fluc 400m/d (2 wk) | 16 | 3/16 | NR |
| Chotmongkol et al. 2005,Thailan | Prosp, OL,Rand | meA, 30 /, | AmphB 0.7m/k/d (2 wk) | 20 | 2/20 | NR |
|  |  | 60/NM | AmphB 0.7m/k/d + Rifampin 600m/d (2 wk) | 20 | 6/20 | NR |
| Bicanic et al. 2007,South Africa | Prosp, OL,observational | meA, 34/ 22/NM | AmphB 1m/k/d (1 week) | 49 | 16/48 | NR |
|  |  |  | Fluc 400m/d (10 wk) | 5 | 3/4 | NR |
| Pappas et al. 2009,USA, Thailand | Prosp, OL,Rand | meA 37 | AmphB 0.7mg/kg (2 wk) | 47 | NR | NR |
|  |  | /65/meD 18 | AmphB 0.7mg/kg + Fluc 400 (2 wk) | 48 | NR | NR |
|  |  |  | AmphB 0.7mg/kg + Fluc 800 (2 wk) | 48 | NR | NR |
| Hamill et al. 2010,USA, Canada | Prosp,DB,Rand | meA, 38 / 88/NM | AmphB 0.7m/k/d (2 wk) | 87 | 10/87 | 29/87 |
|  |  |  | Lip AmphB 3m/k/d (2 wk) | 180 | 21/180 | 32/180 |
| Nussbaum et al. 2010,Malawi | Prosp, OL, Rand | meD 36 /66 | Fluc 1200m/d (2 wk) | 20 | 11/19 | 2/19 |
|  |  | /meD 21 | Fluc 1200m/d + 5FC 100m/k/d (2 wk) | 21 | 9/21 | 1/21 |
| Loyse et al. 2012,South Africa | Prosp, OL, Rand | meD 34 /49 /meD 37 | AmphB 0.7-1m/k/d + 5FC 100m/k/d (2 wk) | 20 | 6/20 | 3/21 |
|  |  |  | AmphB 0.7-1m/k/d + Fluc 800m/d (2 wk) | 45 | 13/43 | 6/45 |
|  |  |  | AmphB 0.7-1m/k/d + Voriconazole 600m/d (2 wk) | 13 | 3/12 | 1/13 |
| Jarvis et al. 2012,South Africa | Prosp, OL, Rand | meD 32 /44 /meD 27 | AmphB 1m/k/d + 5FC 100m/k/d (2 wk) | 31 | 10/31 | 7/31 |
|  |  |  | IFNg 100ug 2x + AmphB 1m/k/d + 5FC 100m/k/d (2 wk) | 57 | 17/57 | 11/57 |
| Jackson et al. 2012,Malawi | Prosp, OL, Rand | meD 35 /65 /meD 41 | AmphB 1m/k/d (1 week) + Fluc 1200m/d (2 wk) | 20 | NR | 4/20 |
|  |  |  | AmphB 1m/k/d (1 week) + Fluc 1200m/d (2 wk) + 5FC 100m/k/d (2 wk) | 20 | NR | 6/20 |
| Day et al. 2013,Vietnam | Prosp, OL ,Rand | meD 28 /82 | AmphB 1m/k/d (4 wk) | 99 | 44/99 | 2/99 |
|  |  | /meD 18 | AmphB 1m/k/d + 5FC 100m/k/d (2 wk) | 100 | 30/100 | 2/100 |
|  |  |  | AmphB 1m/k/d + Fluc 800m/d (2 wk) | 99 | 33/99 | 2/99 |
| Molloy et al. 2018, United Kingdom | Prosp, OL, Rand | meD 36 /53/meD 25 | AmphB 1m/k/d + 5FC 100m/k/d (2 wk) | 115 | 44/115 | 20/228 |
|  |  |  | AmphB 1m/k/d + Fluc 1200m/d (2 wk) | 114 | 47/114 | NR |
|  |  |  | AmphB 1m/k/d + 5FC 100m/k/d (1 wk), the Fluc 1200 m/d (1wk) | 113 | 27/113 | 17/224 |
|  |  |  | AmphB 1m/k/d + Fluc 1200m/d (1 wk), then Fluc 1200m/d (1 wk) | 111 | 54/111 | NR |
|  |  |  | oral Fluc 1200 m/d+5FC 100 m/k/d (2 wk) | 225 | 79/225 | 11/225 |
| Jarvis et al. 2018,Botswana and Tanzania | Prosp, OL, Rand | meD 38 /54 /meD 32 | L-AmphB 10 mg/kg on D1 (single dose) + Fluc 1200 m/d (2 wk) | 18 | NR | 3/16 |
|  |  |  | L-AmphB 10 mg/kg on D 1 & 5 mg/kg on day 3 (2 doses)+ Fluc 1200 m/d (2 wk) | 20 | NR | 1/18 |
|  |  |  | L-AmphB 10 mg/kg on D 1 and 5 mg/kg on D 3 and 7 (3 doses)+ Fluc 1200 m/d (2 wk) | 20 | NR | 4/18 |
|  |  |  | L-AmphB 3 m/k/d (2 wk) | 21 | NR | 1/17 |
| NCT00885703 | Prosp Rand | NM/NM/NM | AmphB | 108 | 54/108 | 0/108 |
|  |  |  | Fluc_High | 46 | 37/46 | 0/46 |

Abbreviation: 5FC , flucytosine; AmphB, amphotericin B; AmphB_S, short course (only 1week) amphotericin B; Fluc, fluconazole; Fluc_H, high dose ( fluconazole > 800 mg/day ); IFNg, Interferon gamma; Itra, itraconazole; Lip AmphB, liposomal amphotericin B; m/g, mg/day; m/g/k, mg/kg/day; meA, mean; meD, median; OL, open label; NM, no mention; NR, not reported; Prosp, Prospective; wk, week.

Supplementary Table 7: Characteristics of key findings in the included studies

| Author , Year, Location | key findings |
| --- | --- |
| Larsen et al. 1990, USA | Non-significant mortality reduction with AmphB+5FC compared to fluc (p=0.27) |
| de Gans et al. 1992,Netherlands | Significant increase in complete-response rate with AmphB compared to itra (p=0.009); non-significant decrease in relapse rate with AmphB compared to itra (p=0.22) |
| van der Horst et al. 1997,USA | Non-significant increase in proportion of negative two-week CSF cultures with AmphB+5FC compared to AmphB alone (p=0.06); no significant difference in proportion of patients with stable or proved symptoms (p=0.18) or combined microbiological outcome (p=0.12) |
| Leenders et al. 1997,Netherlands, Australia | Significantly faster rates of CSF culture clearance (p<0.05) with Lip AmphB compared to standard AmphB. |
| Chotmongkol et al. 1997,Thailand | Significantly higher proportion of treatment success with AmphB+5FC + itra compared to AmphB + 5FC alone (p=0.03) |
| Mayanja-Kizza et al. 1998,Uganda | Addition of 5FC significantly increased six-month survival (p=0.022) |
| Brouwer et al. 2004,Thailand | Significantly improved mycological suppression with AmphB+5FC compared to other regimens. |
| Chotmongkol et al. 2005,Thailand | No significant difference in two week or 10-week negative CSF between arms; addition of rifampin produced non-significant increase in mortality |
| Bicanic et al. 2007,South Africa | Significantly improved mycological suppression with AmphB compared to fluc (p=0.001), maintained after controlling for baseline CSF IFNg and baseline colony forming units; no significant survival difference at two or 10 weeks, but improved mean survival time with AmphB compared to fluc (p=0.03) |
| Pappas et al. 2009,USA, Thailand | No significant difference in toxicities between arms; no significant difference in combined clinical mycologic success between arms, although trend towards greater success in combination therapy arms |
| Hamill et al. 2010,USA, Canada | Fewer infusion related reactions among patients receiving liposomal AmphB compared to standard AmphB (p<0.001); patients receiving Lip AmphB 3mg/kg/d experienced less nephrotoxicity than patients receiving standard AmphB (p=0.003) |
| Nussbaum et al. 2010,Malawi | Addition of 5FC resulted in significantly higher (more negative) mycological suppression (p<0.001); addition of 5FC produced significantly lower mortality at two weeks (p=0.05) but not at 10 weeks (p=0.25) |
| Loyse et al. 2012,South Africa | No significant differences in mycological suppression between arms; no significant differences in mortality between arms |
| Jarvis et al. 2012,South Africa | Significantly improved mycological suppression in two-dose (p=0.02) and six-dose (p=0.006) compared to AmphB + 5FC alone; no significant difference in mortality between groups |
| Jackson et al. 2012,Malawi | Authors combined results with analysis from Nussbaum 2010: addition of 5FC significantly reduced mortality at 2 weeks (p=0.05), but not at 10 weeks; no significant mortality benefit from addition of AmphB. |
| Day et al. 2013,Vietnam | Significant reduction in mortality with AmphB + 5FC compared to AmphB alone at 10 weeks (p=0.04) and 6 months (p=0.01). No significant different in mortality between AmphB + fluc versus AmpgB alone or between AmpgB + fluc and AmphB + 5FC. |
| Molloy et al. 2018, United Kingdom | As a partner drug with AmphB, 5FC was superior to fluc (71 deaths [31.1%] vs. 101 deaths [45.0%]; hazard ratio for death at 10 weeks, 0.62; 95% confidence interval [CI], 0.45 to 0.84; P = 0.002). One week of AmphB plus 5FC was associated with the lowest 10-week mortality (24.2%; 95% CI, 16.2 to 32.1). |
| Jarvis et al. 2018,Botswana and Tanzania | The mortality difference between the single-dose and control arms was 6.4% (95% CI,–21% to 34%). |
| NCT00885703 | no significant differences in mortality between arms |

Abbreviation: 5FC , flucytosine; AmphB, amphotericin B; Fluc, fluconazole; IFNg, Interferon gamma; Itra, itraconazole; Lip AmphB, liposomal amphotericin B

Supplementary Table 8A League table of the early mortality rate

network (lower-left portion) meta-analysis results are presented as estimate effect sizes for the outcome of early mortality in patients with NM. Interventions are reported in order of mean ranking of early mortality, and outcomes are expressed as Odds ratio (OR) (95% confidence intervals).

For the network meta-analysis (NMA), OR of more than 1 indicate that the treatment specified in the column got better early mortality than that specified in the row.

Bold results marked with * indicate statistical significance.

Abbreviation : AmphB, amphotericin B; AmphB_S, short course (only 1week) amphotericin B; Azole_H, high dose ( > 800 mgday )fluconazole; 5FC , flucytosine; Lip AmphB, liposomal amphotericin B

Supplementary Table 8B League table of the late mortality rate

network (lower-left portion) meta-analysis results are presented as estimate effect sizes for the outcome of late mortality in patients with NM. Interventions are reported in order of mean ranking of late mortality, and outcomes are expressed as Odds ratio (OR) (95% confidence intervals).

For the network meta-analysis (NMA), OR of more than 1 indicate that the treatment specified in the column got better late mortality than that specified in the row.

Bold results marked with * indicate statistical significance.

Abbreviation : AmphB, amphotericin B; AmphB_S, short course (only 1week) amphotericin B; Azole_H, high dose ( > 800 mgday )fluconazole; 5FC , flucytosine; Lip AmphB, liposomal amphotericin B

Supplementary Table8C League table of the mycological suppression

network (lower-left portion) meta-analysis results are presented as estimate effect sizes for the outcome of microbiological activity in patients with NM. Interventions are reported in order of mean ranking of mycological suppression, and outcomes are expressed as Odds ratio (OR) (95% confidence intervals).

For the network meta-analysis (NMA), OR of more than 1 indicate that the treatment specified in the column got better microbiological activity than that specified in the row.

Bold results marked with * indicate statistical significance.

Abbreviation : AmphB, amphotericin B; AmphB_S, short course (only 1week) amphotericin B; Azole_H, high dose ( > 800 mgday )fluconazole; 5FC , flucytosine; Lip AmphB, liposomal amphotericin B

Supplementary Table 8D League table of the hepatic adverse reaction

network (lower-left portion) meta-analysis results are presented as estimate effect sizes for the outcome of adverse reaction in patients with NM. Interventions are reported in order of mean ranking of adverse reaction, and outcomes are expressed as Odds ratio (OR) (95% confidence intervals).

For the network meta-analysis (NMA), OR of more than 1 indicate that the treatment specified in the column got better adverse reaction than that specified in the row.

Bold results marked with * indicate statistical significance.

Abbreviation: AmphB, amphotericin B; AmphB_S, short course (only 1week) amphotericin B; Azole_H, high dose ( > 800 mgday )fluconazole; CI: confidence interval; 5FC , flucytosine; LipAmB, liposomal amphotericin B; NMA: network meta-analysis; RCT: randomized controlled trial; RR: rate ratio.

Supplementary Table 9 SUCRA of the outcomes of individual interventions

| early mortality |  | late mortality |  | mycological suppression |  | adverse reaction |  |
| --- | --- | --- | --- | --- | --- | --- | --- |
| Treatment | SUCRA | Treatment | SUCRA | Treatment | SUCRA | Treatment | SUCRA |
| AmphB | 21.2 | AmphB | 32.6 | AmphB | 41 | AmphB | 21.2 |
| AmphB+5FC | 52.4 | AmphB+5FC | 56 | AmphB+5FC | 67 | AmphB+5FC | 52.4 |
| AmphB+Azole | 36.7 | AmphB+Azole | 32.3 | AmphB+Azole | 33.2 | AmphB+Azole | 36.7 |
| AmphB+others | 47.7 | AmphB+others | 47.3 | AmphB+others | 47.3 | AmphB+others | 47.7 |
| AmphB+5FC+Azole | 88.3 | AmphB+5FC+Azole | 39.8 |  |  |  |  |
| LipAmB | 46 | LipAmB | 26.9 | LipAmB | 50.3 | LipAmB | 88.3 |
| Azole | 40 | Azole | 55.7 |  |  | Azole | 46 |
| Azole+5FC | 72.6 | Azole+5FC | 58.9 |  |  |  |  |
| AmphB+5FC+others | 65.1 |  |  | AmphB+5FC+others | 68.8 | AmphB+5FC+others | 40 |
| LipAmB+Azole | 29.9 | LipAmB+Azole | 39.7 | LipAmB+Azole | 1.2 | LipAmB+Azole | 72.6 |
| AmphB_S | 76.5 | AmphB_S | 76.3 |  |  |  |  |
| Azole_H | 33.5 | Azole_H | 94.1 | Azole_H | 64.8 | Azole_H | 65.1 |
| AmphB+Azole_H | 33.5 | AmphB+Azole_H | 49.8 | AmphB+Azole_H | 62.1 | AmphB+Azole_H | 29.9 |
| 5FC+Azole_H | 59.4 | 5FC+Azole_H | 54.9 | 5FC+Azole_H | 20.4 | 5FC+Azole_H | 76.5 |
| others+Azole_H | 19.3 | others+Azole_H | 19.2 | others+Azole_H | 94 | others+Azole_H | 33.5 |
| 5FC+AmphB_S+Azole_H | 52.4 | 5FC+AmphB_S+Azole_H | 34.3 |  |  | 5FC+AmphB_S+Azole_H | 33.5 |
| 5FC+AmphB_S | 75.7 | 5FC+AmphB_S | 82.3 |  |  | 5FC+AmphB_S | 59.4 |

Sorted by preference rankings, with superior treatments (those producing the best preferent according to outcome of mortality, mycological suppression, and adverse event) listed first

The SUCRA rankings here indicated that the AmphB+5FC+Azole were most likely to result in the least incidence of early mortality rate.

Abbreviation: AmphB, amphotericin B; AmphB_S, short course (only 1week) amphotericin B; AR, adverse reaction; Azole_H, high dose ( > 800 mgday )fluconazole; CI: confidence interval; ES: effect size; 5FC , flucytosine; LipAmB, liposomal amphotericin B; NMA: network meta-analysis; RCT: randomized controlled trial; RR: rate ratio; SUCRA: surface under the cumulative ranking curve.

Supplementary Table 10 Inconsistency of different intervention

1. design-by-treatment inconsistency and loop inconsistency

| Inconsistency model | chi2 | Prob>chi2 |
| --- | --- | --- |
| Early mortality | | |
| design-by-treatment | 13.03 | 0.16 |
| loop inconsistency | 7.12 | 0.13 |
| Late mortality | | |
| design-by-treatment | 11.43 | 0.25 |
| loop inconsistency | 3.02 | 0.55 |
| microbiological activity | | |
| design-by-treatment |  |  |
| loop inconsistency |  |  |
| hepatic adverse reaction | | |
| design-by-treatment | 13.03 | 0.16 |
| loop inconsistency | 7.12 | 0.13 |

(b-1) site splitting inconsistency for early mortality rate

|  |  |  | early mortality rate | | | | |  |  |  |  |
| --- | --- | --- | --- | --- | --- | --- | --- | --- | --- | --- | --- |
|  |  | all, tau | | | all, tau nosymmetric | | |  |  | Symbols | |
|  |  |  | P>z | tau |  | P>z | tau |  |  | A | AmphB |
| A | B |  | 0.94 | 0.58 |  | 0.70 | 0.56 |  |  | B | AmphB+5FC |
| A | C |  | 0.18 | 0.06 |  | 0.05 | 0.00 |  |  | C | AmphB+Azole |
| A | D |  | 0.11 | 0.35 |  | 0.53 | 0.52 |  |  | D | AmphB+others |
| A | F | * | 1.00 | 0.40 | * | 1.00 | 0.40 |  |  | E | AmphB+5FC+Azole |
| A | L |  | 0.14 | 0.33 |  | 0.14 | 0.33 |  |  | F | LipAmphB |
| A | M |  | 0.28 | 0.49 |  | 0.38 | 0.50 |  |  | G | Azole |
| B | C | * | 0.92 | 0.55 | * | 0.91 | 0.53 |  |  | H | Azole+5FC |
| B | D |  | 0.73 | 0.57 |  | 0.03 | 0.30 |  |  | I | AmphB+5FC+others |
| B | E | * | 1.00 | 0.40 |  | . | . |  |  | J | LipAmphB+Azole |
| B | G | * | 1.00 | 0.40 | * | 1.00 | 0.40 |  |  | K | AmphB_S |
| B | I | * | 1.00 | 0.40 |  | . | . |  |  | L | Azole_H |
| B | M |  | 0.47 | 0.50 |  | 0.45 | 0.45 |  |  | M | AmphB+Azole_H |
| B | N | * | 0.90 | 0.59 | * | 0.14 | 0.33 |  |  | N | 5FC+Azole_H |
| B | O | * | 0.49 | 0.52 | * | 1.00 | 0.40 |  |  | O | Azole_H+others |
| B | Q | * | 0.49 | 0.52 |  | . | . |  |  | P | 5FC+AmphB_S+Azole_H |
| C | D |  | 0.04 | 0.00 |  | 0.36 | 0.41 |  |  | Q | 5FC+AmphB_S |
| C | M |  | 0.71 | 0.47 |  | 0.45 | 0.45 |  |  |  |  |
| C | N | * | 0.02 | 0.00 | * | 0.14 | 0.33 |  |  |  |  |
| C | O | * | 0.05 | 0.00 | * | 1.00 | 0.40 |  |  |  |  |
| C | Q | * | 0.05 | 0.00 |  | . | . |  |  |  |  |
| D | M |  | 0.46 | 0.50 |  | 0.38 | 0.50 |  |  |  |  |
| F | J | * | 1.00 | 0.40 |  | . | . |  |  |  |  |
| G | H | * | 1.00 | 0.40 |  | . | . |  |  |  |  |
| G | K | * | 1.00 | 0.40 |  | . | . |  |  |  |  |
| L | N |  | 0.14 | 0.33 |  | 0.14 | 0.33 |  |  |  |  |
| N | O | * | 0.14 | 0.33 | * | 1.00 | 0.40 |  |  |  |  |
| N | Q | * | 0.14 | 0.33 |  | . | . |  |  |  |  |
| O | P | * | 1.00 | 0.40 |  | . | . |  |  |  |  |
| O | Q | * | 1.00 | 0.40 |  | . | . |  |  |  |  |

(b-2) site splitting inconsistency for late mortality rate

|  |  |  | late mortality rate | | | | |  |  |  |
| --- | --- | --- | --- | --- | --- | --- | --- | --- | --- | --- |
|  |  | all, tau | | | all, tau nosymmetric | | |  | Symbols | |
|  |  |  | P>z | tau |  | P>z | tau |  | A | AmphB |
| A | B |  | 0.05 | 0.00 |  | 0.08 | 0.00 |  | B | AmphB+5FC |
| A | C |  | 0.54 | 0.22 |  | 0.45 | 0.26 |  | C | AmphB+Azole |
| A | D |  | 0.10 | 0.10 |  | 0.38 | 0.25 |  | D | AmphB+others |
| A | E | * | 1.00 | 0.23 | * | 1.00 | 0.23 |  | E | AmphB+5FC+Azole |
| A | K |  | 0.28 | 0.12 |  | 0.28 | 0.12 |  | F | LipAmphB |
| A | L |  | 0.08 | 0.00 |  | 0.90 | 0.30 |  | G | Azole |
| B | C | * | 0.85 | 0.35 | * | 0.39 | 0.34 |  | H | Azole+5FC |
| B | D |  | 0.53 | 0.28 |  | 0.06 | 0.07 |  | I | LipAmphB+Azole |
| B | F | * | 1.00 | 0.23 | * | 1.00 | 0.23 |  | J | AmphB_S |
| B | H | * | 1.00 | 0.23 |  | . | . |  | K | Azole_H |
| B | L |  | 0.83 | 0.29 |  | 0.55 | 0.28 |  | L | AmphB+Azole_H |
| B | M | * | 0.10 | 0.00 | * | 0.28 | 0.12 |  | M | 5FC+Azole_H |
| B | N | * | 0.12 | 0.00 | * | 1.00 | 0.23 |  | N | Azole_H+others |
| B | P | * | 0.12 | 0.00 |  | . | . |  | O | 5FC+AmphB_S+Azole_H |
| C | D |  | 0.12 | 0.16 |  | 0.64 | 0.26 |  | P | 5FC+AmphB_S |
| C | L |  | 0.40 | 0.31 |  | 0.55 | 0.28 |  |  |  |
| C | M | * | 0.91 | 0.31 | * | 0.28 | 0.12 |  |  |  |
| C | N | * | 0.45 | 0.26 | * | 1.00 | 0.23 |  |  |  |
| C | P | * | 0.45 | 0.26 |  | . | . |  |  |  |
| D | L |  | 0.74 | 0.29 |  | 0.90 | 0.30 |  |  |  |
| E | I | * | 1.00 | 0.23 |  | . | . |  |  |  |
| F | G | * | 1.00 | 0.23 |  | . | . |  |  |  |
| F | J | * | 1.00 | 0.23 |  | . | . |  |  |  |
| K | M |  | 0.28 | 0.12 |  | 0.28 | 0.12 |  |  |  |
| M | N | * | 0.28 | 0.12 | * | 1.00 | 0.23 |  |  |  |
| M | P | * | 0.28 | 0.12 |  | . | . |  |  |  |
| N | O | * | 1.00 | 0.23 |  | . | . |  |  |  |
| N | P | * | 1.00 | 0.23 |  | . | . |  |  |  |
|  |  |  |  |  |  |  |  |  |  |  |

(b-3) site splitting inconsistency for microbiological activity

|  |  |  | microbiological activity | | | | |  |  |  |
| --- | --- | --- | --- | --- | --- | --- | --- | --- | --- | --- |
|  |  | all, tau | | | all, tau nosymmetric | | |  | Symbols | |
|  |  |  | P>z | tau |  | P>z | tau |  | A | AmphB |
| A | B |  | 0.01 | 0.00 |  | 0.07 | 0.35 |  | B | AmphB+5FC |
| A | C |  | 0.33 | 0.44 |  | 0.09 | 0.35 |  | C | AmphB+Azole |
| A | D |  | 0.23 | 0.44 |  | 0.43 | 0.55 |  | D | AmphB+others |
| A | E |  | . | . |  | . | . |  | E | LipAmphB |
| A | G |  | 0.52 | 0.54 |  | 0.52 | 0.54 |  | F | Azole |
| A | H |  | 0.18 | 0.41 |  | 0.70 | 0.61 |  | G | Azole+5FC |
| B | C | * | 0.04 | 0.00 |  | . | . |  | H | AmphB+5FC+others |
| B | D |  | 0.80 | 0.58 |  | 0.14 | 0.42 |  | I | LipAmphB+Azole |
| B | F | * | 1.00 | 0.52 |  | . | . |  | J | AmphB_S |
| B | H | * | 0.04 | 0.00 |  | . | . |  | K | Azole_H |
| B | I | * | 0.01 | 0.00 | * | 0.52 | 0.54 |  | L | AmphB+Azole_H |
| B | J | * | 0.01 | 0.00 |  | . | . |  | M | 5FC+Azole_H |
| B | K | * | 0.01 | 0.00 |  | . | . |  | N | Azole_H+others |
| C | D |  | 0.18 | 0.41 |  | 0.67 | 0.54 |  | O | 5FC+AmphB_S+Azole_H |
| C | I | * | 0.54 | 0.59 | * | 0.52 | 0.54 |  | P | 5FC+AmphB_S |
| C | J | * | 0.09 | 0.35 |  | . | . |  |  |  |
| C | K | * | 0.09 | 0.35 |  | . | . |  |  |  |
| D | H |  | 0.50 | 0.59 |  | 0.70 | 0.61 |  |  |  |
| G | I |  | 0.52 | 0.54 |  | 0.52 | 0.54 |  |  |  |
| I | J | * | 0.52 | 0.54 |  | . | . |  |  |  |
| I | K | * | 0.52 | 0.54 |  | . | . |  |  |  |
| J | K |  | . | . |  | . | . |  |  |  |

(b-4) site splitting inconsistency for hepatic adverse reaction

|  |  |  |  | Hepatic adverse reaction | | | | |  |  |  |
| --- | --- | --- | --- | --- | --- | --- | --- | --- | --- | --- | --- |
|  |  |  | all, tau | | | all, tau nosymmetric | | |  | Symbols | |
|  |  |  |  | P>z | tau |  | P>z | tau |  | A | AmphB |
|  | A | B |  | 0.88 | 0.00 |  | 0.95 | 0.00 |  | B | AmphB+5FC |
|  | A | E | * | 1.00 | 0.00 | * | 1.00 | 0.00 |  | C | AmphB+Azole |
|  | A | I |  | 0.62 | 0.00 |  | 0.62 | 0.00 |  | D | AmphB+others |
|  | A | J |  | 0.60 | 0.00 |  | 0.72 | 0.00 |  | E | LipAmphB |
|  | B | C | * | 0.62 | 0.00 |  | . | . |  | F | Azole |
|  | B | D | * | 0.72 | 0.00 |  | . | . |  | G | AmphB+5FC+others |
|  | B | F | * | 1.00 | 0.00 |  | . | . |  | H | LipAmphB+Azole |
|  | B | G | * | 1.00 | 0.00 |  | . | . |  | I | Azole_H |
|  | B | J | * | 0.62 | 0.00 |  | . | . |  | J | AmphB+Azole_H |
|  | B | K | * | 0.62 | 0.00 | * | 0.62 | 0.00 |  | K | 5FC+Azole_H |
|  | B | L | * | 0.62 | 0.00 | * | 1.00 | 0.00 |  | L | Azole_H+others |
|  | B | N | * | 0.62 | 0.00 |  | . | . |  | M | 5FC+AmphB_S+Azole_H |
|  | C | K | * | 0.62 | 0.00 | * | 0.62 | 0.00 |  | N | 5FC+AmphB_S |
|  | C | L | * | 1.00 | 0.00 | * | 1.00 | 0.00 |  |  |  |
|  | C | N |  | . | . |  | . | . |  |  |  |
|  | D | J | * | 0.72 | 0.00 | * | 0.72 | 0.00 |  |  |  |
|  | E | H | * | 1.00 | 0.00 |  | . | . |  |  |  |
|  | I | K |  | 0.62 | 0.00 |  | 0.62 | 0.00 |  |  |  |
|  | K | L | * | 0.62 | 0.00 | * | 1.00 | 0.00 |  |  |  |
|  | K | N | * | 0.62 | 0.00 |  | . | . |  |  |  |
|  | L | M | * | 1.00 | 0.00 |  | . | . |  |  |  |
|  | L | N | * | 1.00 | 0.00 |  | . | . |  |  |  |
