# Supplementary material for: Efficacy of induction regimens for cryptococcal meningitis in HIV-infected adults: a systematic review and network meta-analysis
Source: Sci Rep. 2021 Apr 21;11:8565. doi: 10.1038/s41598-021-87726-6 (PMC8060388; doi:10.1038/s41598-021-87726-6)
Supplement: Supplementary file 2 — Supplementary Figures. [file 41598_2021_87726_MOESM2_ESM.docx]

Category of paper: Review

Article title: Re-appraisal of effective, and tolerable induction regimens for managing cryptococcal meningitis in HIV-infected adults: a systematic review and network meta-analysis

Authors details

Chang-Hua Chen ^a,b,c,d^, Hua Li^e, @^, Hsien-Meng Chen^a^, Yu-Min Chen ^f^, Yu-Jun Chang ^g^, Pao-Yen Lin ^h, i,^ Chih-Wei Hsu ^h, j^, Ping-Tao Tseng ^k^, Kai-Huang Lin^l,^ Yu-Kang Tu ^m,n,*^

Affiliation:

^a^ Division of Infectious Disease, Department of Internal Medicine, Changhua Christian Hospital, Changhua, 500,Taiwan;

^b^ Center for Infection Prevention and Control, Changhua Christian Hospital, Changhua, 500, Taiwan ;

^c^ Ph.D. Program in Translational Medicine, National Chung Hsing University, Taichung County, 402, Taiwan ;

^d^ Rong Hsing Research Center For Translational Medicine, National Chung Hsing University, Taichung County, 402, Taiwan ;

^e^ Institute of Epidemiology & Preventive Medicine, College of Public Health, National Taiwan University, Taipei, Taiwan

^f^ Department of Pharmacy, Changhua Christian Hospital, Changhua, Taiwan

^g^ Epidemiology and Biostatistics Center, Changhua Christian Hospital, Changhua, Taiwan

^h^ Department of Psychiatry, Kaohsiung Chang Gung Memorial Hospital, Kaohsiung, Taiwan

^i^ Chang Gung University College of Medicine, Kaohsiung, Taiwan

^j^ Department of Computer Science and Information Engineering, National Cheng Kung University, Tainan, Taiwan

^k^ WinShine Clinics in Specialty of Psychiatry, Kaohsiung City, Taiwan

^l^ Division of Critical Care Medicine, Department of Internal Medicine, Changhua Christian Hospital, Changhua, 500,Taiwan;

^m^Institute of Epidemiology & Preventive Medicine, College of Public Health, National Taiwan University, Taipei, Taiwan

^n^ Department of Dentistry, National Taiwan University Hospital, Taipei, Taiwan

^@^: equal contribution

Corresponding author : Yu-Kang Tu, Institute of Epidemiology & Preventive Medicine, College of Public Health, National Taiwan University, Taipei, Taiwan; e-mail: yukangtu@ntu.edu.tw

**Figure legends for Supplementary Figures List**

Supplementary Figure 1A-1B Network structure of network meta-analysis of different outcomes for Cryptococcal meningitis in HIV patients

Supplementary Figure 2A-2B: forest plot of network meta-analysis of different outcomes for Cryptococcal meningitis in HIV patients

Supplementary Figure 3 overview of risk of bias

Supplementary Figure 4 A-4H Funnel plot of network meta-analysis of different outcomes for Cryptococcal meningitis in HIV patients

**Supplementary Figure 1A-1B: Network structure of network meta-analysis of different outcomes for Cryptococcal meningitis in HIV patients**

The lines between nodes represent direct comparisons in various trials, and the size of each circle is proportional to the size of the population involved in each specific treatment. The thickness of the lines is proportional to the number of trials connected to the network.

Abbreviation:

AmphB, amphotericin B; AmphB_S, short course (only 1week) amphotericin B; Azole_H, high dose ( > 800 mgday )fluconazole; 5FC , flucytosine; Lip AmphB, liposomal amphotericin B;

Supplementary Figure 1A Network structure of network meta-analysis of mycological suppression for Cryptococcal meningitis in HIV patients

Supplementary Figure 1B Network structure of network meta-analysis of hepatic adverse reaction for Cryptococcal meningitis in HIV patients

Supplementary Figure 2A-2B: forest plot of network meta-analysis of different outcomes for Cryptococcal meningitis in HIV patients

Abbreviation:

AmphB, amphotericin B; AmphB_S, short course (only 1week) amphotericin B; Azole_H, high dose ( > 800 mgday )fluconazole; 5FC , flucytosine; Lip AmphB, liposomal amphotericin B


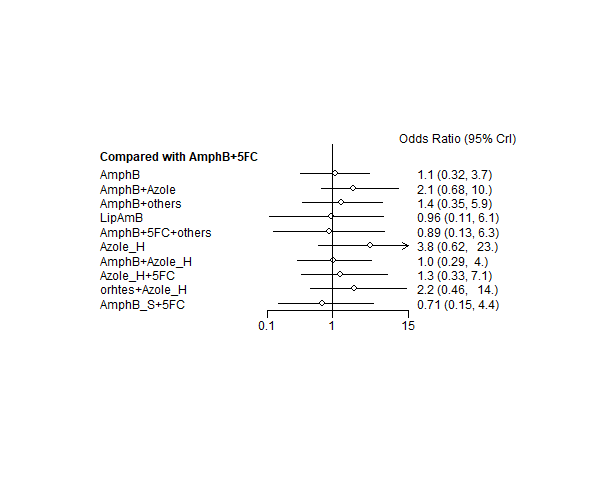


Supplementary Figure 2A forest plot of network meta-analysis of mycological suppression for Cryptococcal meningitis in HIV patients.

Notes: the left of the null axis at 1 favor the lower mycological suppression and those to the right favor the higher mycological suppression.


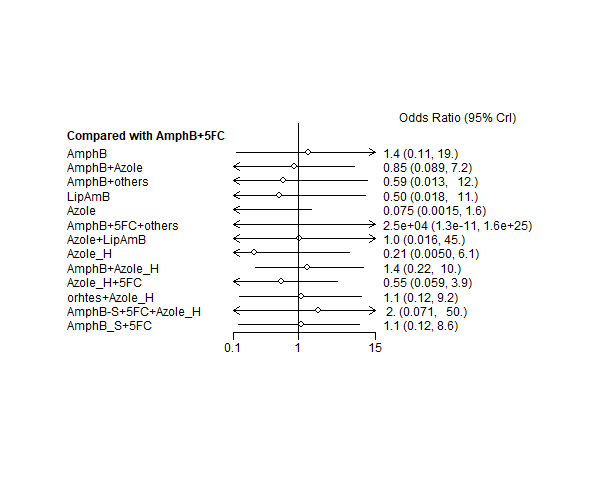


Supplementary Figure 2B forest plot of network meta-analysis of hepatic adverse reaction for Cryptococcal meningitis in HIV patients.

Notes: the left of the null axis at 1 favor the lower hepatic adverse reaction and those to the right favor the higher hepatic adverse reaction.

Supplementary Figure 3 overview of risk of bias

Supplementary Figure 4 A-4H Funnel plot of network meta-analysis of different outcomes for Cryptococcal meningitis in HIV patients

Abbreviation

AmphB, amphotericin B; AmphB_S, short course (only 1week) amphotericin B; Azole_H, high dose ( > 800 mgday )fluconazole; 5FC , flucytosine; Lip AmphB, liposomal amphotericin B;

Symbol: A:AmphB,B:AmphB+5FC,C:AmphB+Azole,D:AmphB+others,E:AmphB+5FC+Azole,F:LipAmphB,G:Azole,H:Azole+5FC,I:AmphB+5FC+others,J:LipAmphB+Azole,K:AmphB_S,L:Azole_H,M:AmphB+Azole_H,N:5FC+Azole_H,O:Azole_H+others,P:5FC+AmphB_S+Azole_H,Q:5FC+AmphB_S.


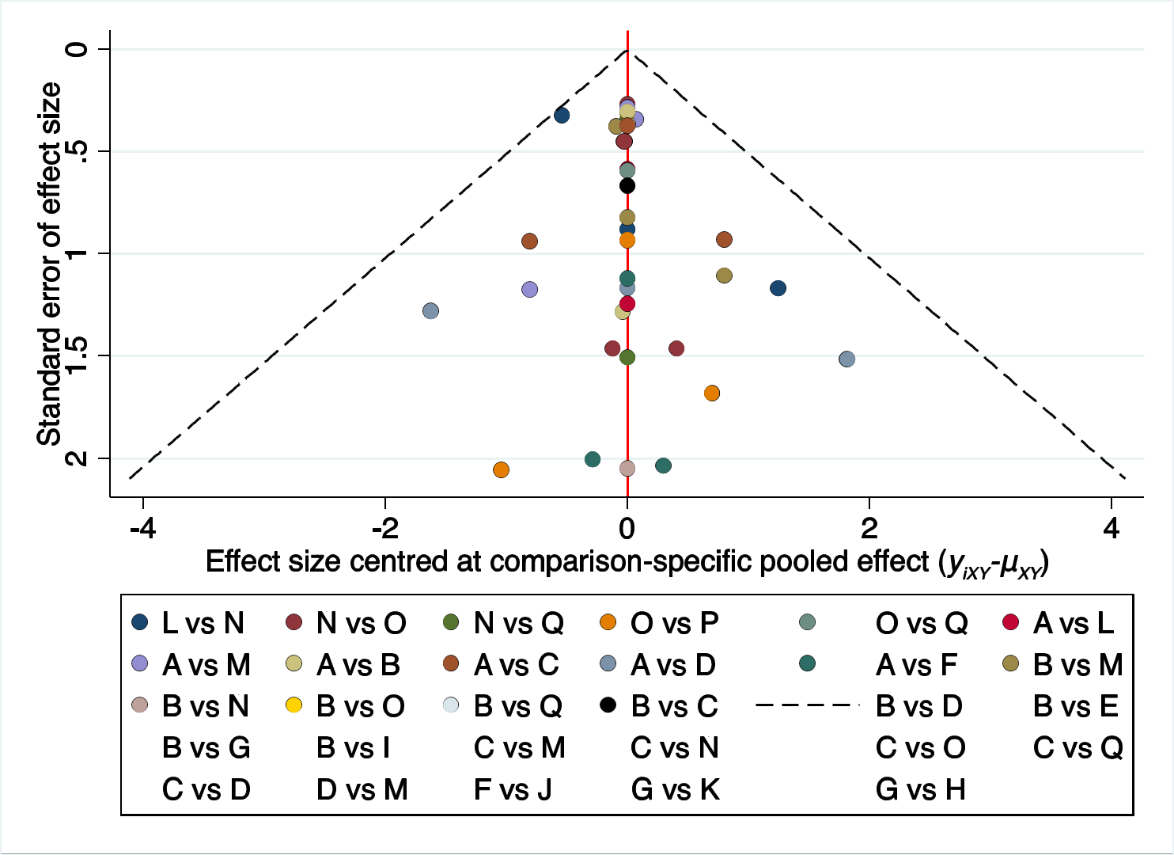
Figure 4A Funnel plot of network meta-analysis of early mortality rate for Cryptococcal meningitis in HIV patients


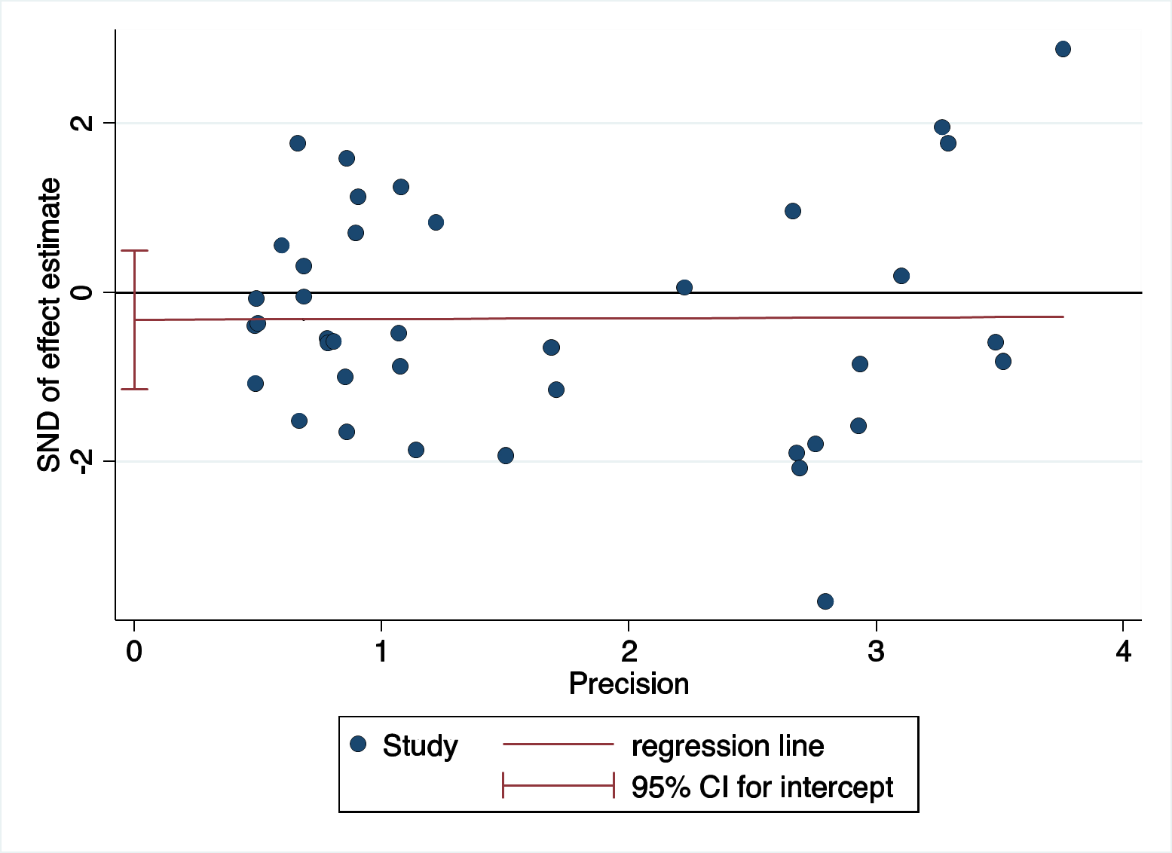


Supplementary Figure 4B Egger's regression of network meta-analysis of early mortality rate for Cryptococcal meningitis in HIV patients


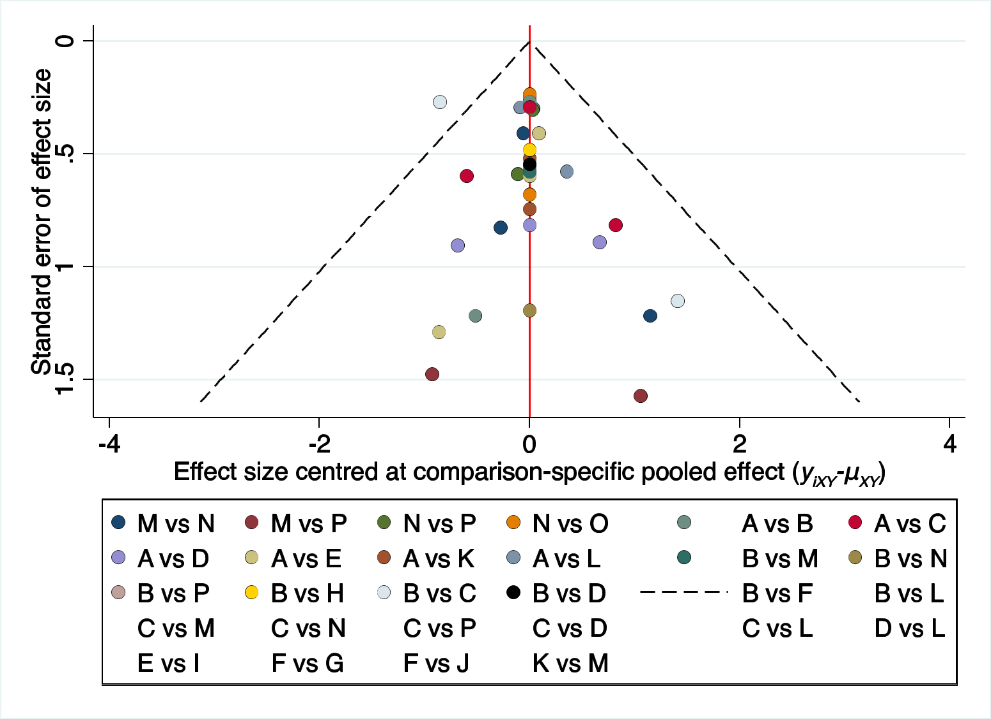
Supplementary Figure 4C Funnel plot of network meta-analysis of late mortality rate for Cryptococcal meningitis in HIV patients


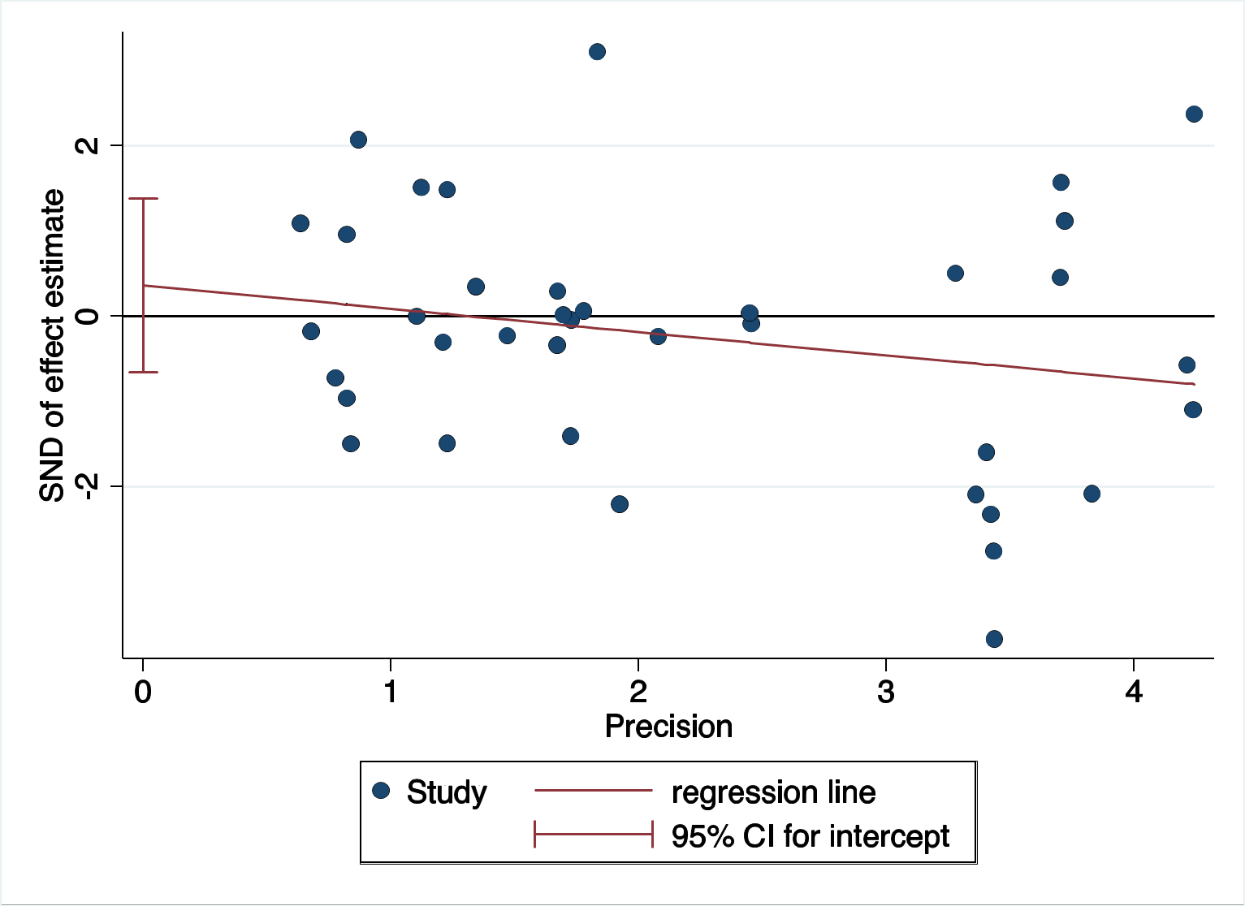


Supplementary Figure 4D Egger's regression of network meta-analysis of late mortality rate for Cryptococcal meningitis in HIV patients


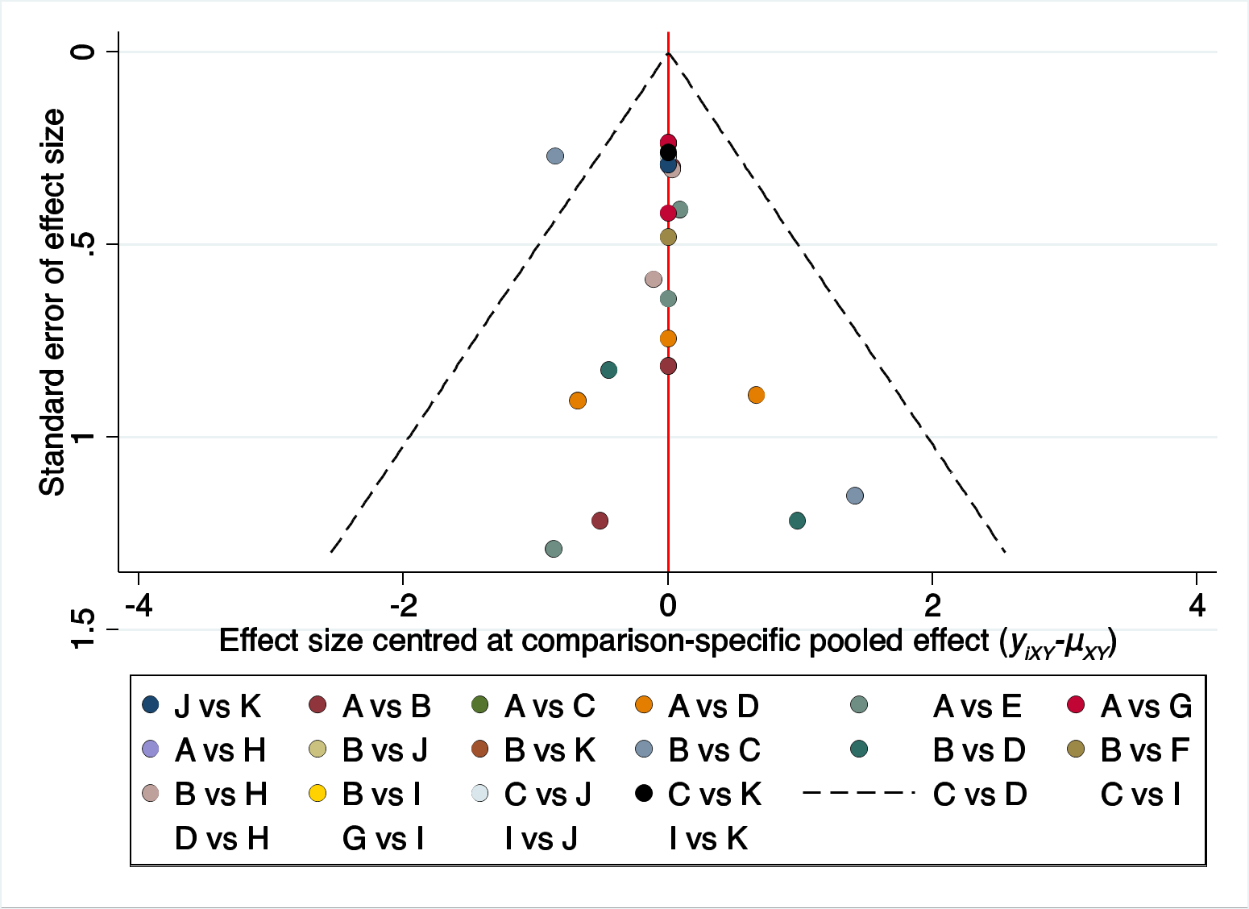
Supplementary Figure 4E Funnel plot of network meta-analysis of mycological suppression for Cryptococcal meningitis in HIV patients


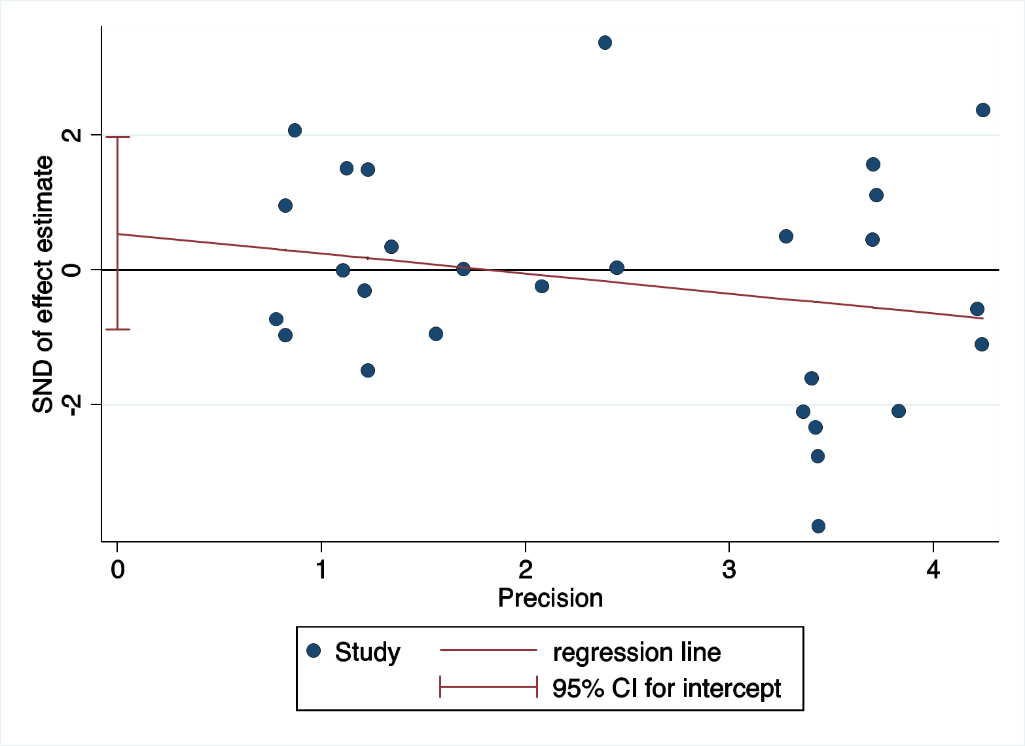
Supplementary Figure 4F Egger's regression of network meta-analysis of mycological suppression for Cryptococcal meningitis in HIV patients


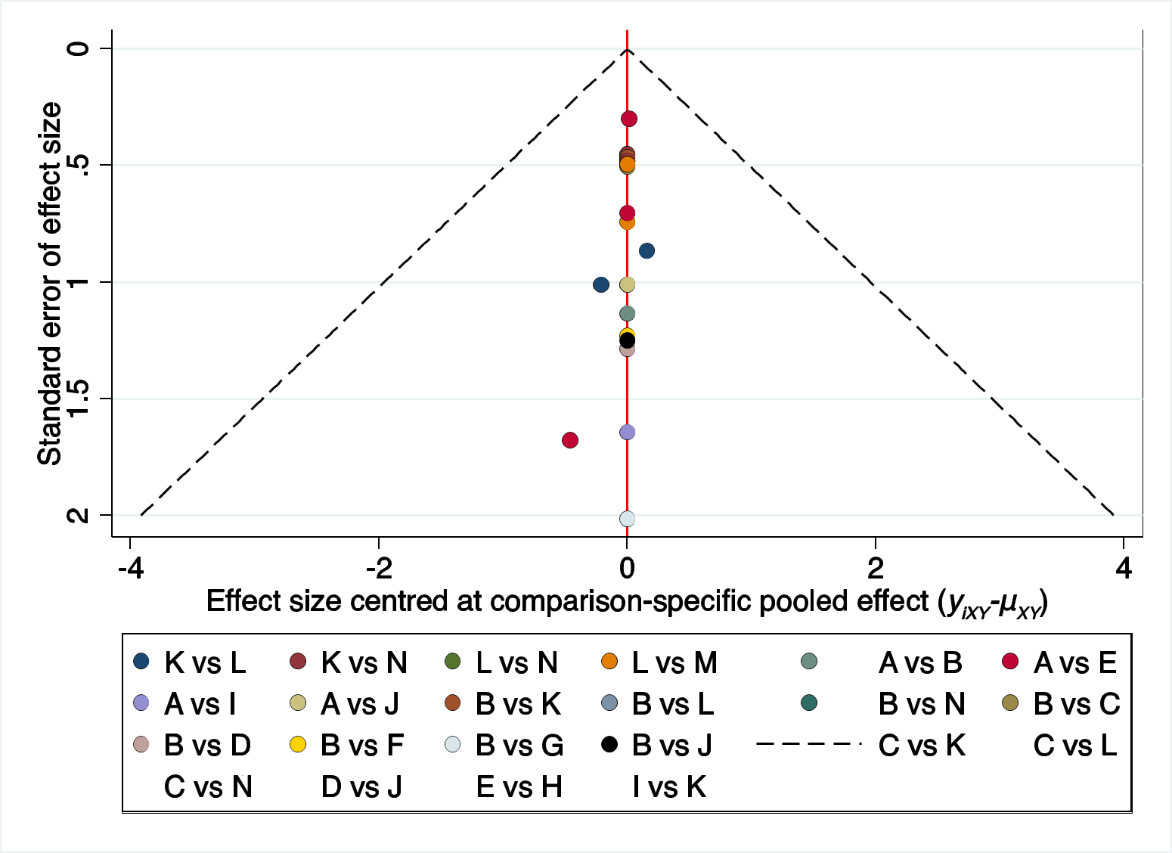


Supplementary Figure 4G Funnel plot of network meta-analysis of hepatic adverse reaction for Cryptococcal meningitis in HIV patients


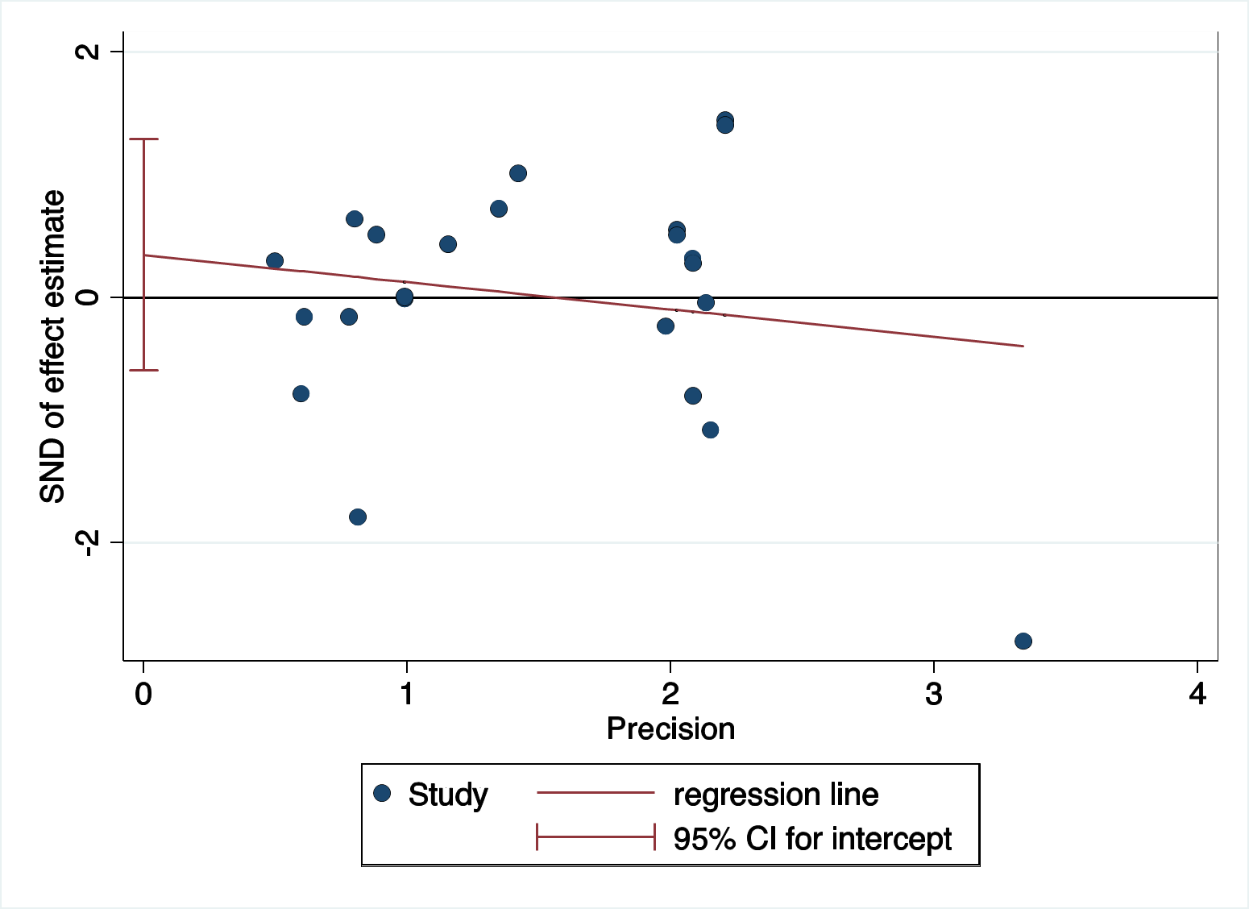
Supplementary Figure 4H Egger's regression of network meta-analysis of hepatic adverse reaction for Cryptococcal meningitis in HIV patients
